# Mapping‐by‐sequencing in complex polyploid genomes using genic sequence capture: a case study to map yellow rust resistance in hexaploid wheat

**DOI:** 10.1111/tpj.13204

**Published:** 2016-07-18

**Authors:** Laura‐Jayne Gardiner, Pauline Bansept‐Basler, Lisa Olohan, Ryan Joynson, Rachel Brenchley, Neil Hall, Donal M. O'Sullivan, Anthony Hall

**Affiliations:** ^1^ Institute of Integrative BiologyUniversity of LiverpoolCrown StreetLiverpoolUK; ^2^ SyngentaFerme de Moyencourt78910OrgerusFrance; ^3^ School of Agriculture, Policy and DevelopmentUniversity of ReadingPO Box 237, WhiteknightsReadingRG6 6ARUK; ^4^ Present address: SyngentaFerme de Moyencourt78910OrgerusFrance

**Keywords:** mapping‐by‐sequencing, wheat, target enrichment, genomics, next generation

## Abstract

Previously we extended the utility of mapping‐by‐sequencing by combining it with sequence capture and mapping sequence data to pseudo‐chromosomes that were organized using wheat–*Brachypodium* synteny. This, with a bespoke haplotyping algorithm, enabled us to map the flowering time locus in the diploid wheat *Triticum monococcum* L. identifying a set of deleted genes (Gardiner *et al*., 2014). Here, we develop this combination of gene enrichment and sliding window mapping‐by‐synteny analysis to map the *Yr6* locus for yellow stripe rust resistance in hexaploid wheat. A 110 MB NimbleGen capture probe set was used to enrich and sequence a doubled haploid mapping population of hexaploid wheat derived from an Avalon and Cadenza cross. The *Yr6* locus was identified by mapping to the POPSEQ chromosomal pseudomolecules using a bespoke pipeline and algorithm (Chapman *et al*., 2015). Furthermore the same locus was identified using newly developed pseudo‐chromosome sequences as a mapping reference that are based on the genic sequence used for sequence enrichment. The pseudo‐chromosomes allow us to demonstrate the application of mapping‐by‐sequencing to even poorly defined polyploidy genomes where chromosomes are incomplete and sub‐genome assemblies are collapsed. This analysis uniquely enabled us to: compare wheat genome annotations; identify the *Yr6* locus – defining a smaller genic region than was previously possible; associate the interval with one wheat sub‐genome and increase the density of SNP markers associated. Finally, we built the pipeline in iPlant, making it a user‐friendly community resource for phenotype mapping.

## Significance Statement

It is challenging to apply mapping‐by‐sequencing pipelines to large polyploid genomes such as hexaploid wheat. Here we present a method for the rapid mapping of genes responsible for a phenotype, and demonstrate its utility by mapping a disease resistance gene. We show that our bespoke mapping‐by‐sequencing pipeline, available on iPlant, can be applied to poorly defined polyploid genomes.

## Introduction

Mapping‐by‐sequencing has become a powerful tool for the genetic mapping of mutants and continuous pipeline development has streamlined such analyses in diploid species (Schneeberger *et al*., 2009; Doitsidou *et al*., 2010; Austin *et al*., 2011; Abe *et al*., 2012; Minevich *et al*., 2012). One frequently cited mapping‐by‐sequencing pipeline, SHOREmap, was originally developed for the diploid plant species *Arabidopsis thaliana*. SHOREmap uses sequence data from a phenotyped F2 mapping population to identify mapping intervals containing the phenotype inducing mutation. It scans across a reference sequence and locates a region of homozygosity in the mutant mapping population that is conserved with the mutant parent; however, it does not work for a polyploid dataset (Schneeberger *et al*., 2009; Galvao *et al*., 2012; Hartwig *et al*., 2012).

The application of mapping‐by‐sequencing pipelines to large polyploid genomes such as hexaploid wheat faces three main challenges. The first challenge involves the requirement for whole‐genome re‐sequencing of a phenotyped mapping population. Due to its vast, 17 Gb genome and highly repetitive content, it is still expensive to generate whole‐genome sequence data for wheat (Choulet *et al*., 2010). To reduce this complexity, methods such as transcriptome sequencing (Trick *et al*., 2012), restriction site‐associated DNA sequencing (Baird *et al*., 2008) and targeted enrichment sequencing (Winfield *et al*., 2012), have been proposed. We previously demonstrated the use of a 110 MB NimbleGen SeqCap EZ gene capture probe set to enrich wheat's genic regions prior to sequencing. This greatly reduces the cost associated with sequencing the wheat genome by eliminating repetitive sequence from the analysis while still allowing mapping‐by‐sequencing (Gardiner *et al*., 2014).

The second challenge to apply mapping‐by‐sequencing is the availability of a reference sequence. While analyses have been successful without full chromosome sequences (Mascher *et al*., 2014; Jordan *et al*., 2015), typically utilizing contig or SNP anchoring, the benefit of a continuous sequence for streamlined sliding window mapping‐by‐sequencing analyses is clear and as such many of the current methodologies rely on this information (Galvao *et al*., 2012; Nordstrom *et al*., 2013). For wheat, like many crop species, no finished genome reference sequence is available, and furthermore, for analyses utilizing genic enrichment, mapping across a full reference will be sporadic. However, the current POPSEQ wheat chromosomal pseudomolecules provide a likely adequate reference for mapping‐by‐sequencing analyses where the sub‐genomes of wheat can be distinguished for mapping (Chapman *et al*., 2015). As such, these chromosomal pseudomolecules are implemented in this analysis. However, full genome sequences with distinction between similar sub‐genomes are rare for complex polyploids e.g. sugarcane and cotton.

We have previously demonstrated the utility of combined genic enrichment and a sliding window mapping‐by‐sequencing analysis using synteny, or mapping‐by‐synteny, to map an early flowering mutant earliness *per se* (*Eps‐3A* ^*m*^) in the monocot species *Triticum monococcum* (Gardiner *et al*., 2014; Gawronski *et al*., 2014). In this previous investigation a shotgun analysis of the wheat genome (Brenchley *et al*., 2012) was used to develop the 110 MB NimbleGen SeqCap EZ gene capture probe set. The long‐range order of the short assemblies was approximated using synteny between wheat and *Brachypodium,* that diverged from wheat ~35–40 million years ago (Bossolini *et al*., 2007). This allowed the construction of seven wheat pseudo‐chromosome sequences, collapsed from the 21 chromosome sequences, which could be used as a reference genome for a sliding window mapping‐by‐sequencing analysis. Therefore these collapsed seven pseudo‐chromosomes each represent three sub‐genomes of wheat similarly to our 110 MB capture space, where each probe targets three homoeologous gene copies due to high similarity between them. Here the pseudo‐chromosomes are used as a reference sequence to maximize mapping by eliminating the problems of non‐uniquely mapping reads between the wheat sub‐genomes as reads for all three genomes map to one representative chromosome. These pseudo‐chromosomes also allow us to demonstrate that our bespoke mapping‐by‐sequencing pipeline and algorithm can be applied to poorly defined polyploidy genomes in which chromosomes are incomplete and sub‐genome assemblies are collapsed, using a comparison with the results gained with the POPSEQ wheat chromosomal pseudomolecules as a validation.

The final challenge for sliding window mapping‐by‐sequencing in collapsed wheat pseudo‐chromosomes, combined with sequence capture, is the application to a polyploid genome. Due to high similarity between the sub‐genomes in polyploid wheat, allocation of short sequencing reads to a specific sub‐genome can be problematic. Furthermore, using the collapsed pseudo‐chromosomes as a mapping reference, homozygous homoeologous SNP alleles are seen in only approximately a third of sequencing reads and are therefore more difficult to identify. We previously developed a mapping‐by‐sequencing pipeline and algorithm that prioritized adaptability to polyploid plant species and hypothesized that, with minor adjustments; this method would be easily adaptable to polyploid species (Gardiner *et al*., 2014).

Here, we refine and develop combined genic enrichment and sliding window mapping‐by‐synteny analysis to map the *Yr6* locus that is associated with yellow rust resistance in hexaploid wheat. The *Yr6* locus is reportedly found on wheat chromosome 7B (Li *et al*., 2009b). We use the 110 MB NimbleGen SeqCap EZ gene capture probe set for gene enrichment and develop two timely versions of our wheat pseudo‐chromosome reference sequence for mapping. The first pseudo‐genome, of the two that are detailed here, advances our previously published pseudo‐genome (Gardiner *et al*., 2014) and is organized based on the MIPS Genome Zipper markers plus synteny between wheat and *Brachypodium*. The second pseudo‐genome is based on POPSEQ‐generated markers and derived chromosomal pseudomolecules (Chapman *et al*., 2015). These pseudo‐chromosome sequences will become valuable resources for mapping‐by‐sequencing analyses and the comparison between the two enables a unique analysis of current wheat genome marker resources. The additional mapping analysis using the POPSEQ wheat chromosomal pseudomolecules directly, allows methodology validation for the pseudo‐chromosome sequences.

The mapping population under analysis was a doubled haploid, hexaploid, yellow rust resistant wheat that was derived from a cross between the parental varieties Avalon and Cadenza, previously described in (Mackay *et al*., 2014; Snape *et al*., 2007; see Acknowledgements). Here, the advantage of using a doubled haploid population is that all sites are immediately homozygous in individual progeny lines. Yellow rust or stripe rust, caused by *Puccinia striiformis,* is one of the most important diseases of bread wheat with epidemics often leading to severe wheat yield losses (Chen *et al*., 2014). Although much fungicide application is targeted to the prophylactic control of yellow rust in intensive wheat cultivation in developed countries, in organic and extensive systems and in developing countries generally, the most efficient method of combating yellow rust involves the utilization of resistant cultivars. Frequent emergence of yellow rust races results in resistant wheat cultivars often becoming susceptible after being grown for some period of time (Wellings and McIntosh, 1990; Chen *et al*., 2002). As a result, the search for new yellow rust‐resistance genes and introgression of new resistance genes is carried out on a continual basis (Deng *et al*., 2004). This breeding effort would be significantly enhanced if resistance genes could be fine‐mapped or cloned more efficiently.

Here, with use of our two pseudo‐genomes, we advance our current mapping‐by‐synteny pipeline to: home in on the *Yr6* region of interest, defining a smaller genic region than was previously possible; to associate the region of interest with a specific wheat genome A, B or D and to greatly increase the density of SNP markers associated with the *Yr6* locus with use of both the POPSEQ chromosomal pseudomolecules and a collapsed partial polyploid genome assembly to highlight the suitability of this methodology for poorly defined polyploid genomes. Finally, the entire workflow has been built in iPlant as part of iPlant‐UK allowing researchers with limited bioinformatics experience to re‐run the pipeline in the iPlant collaborative environment (Goff *et al*., 2011).

## Results

### Enrichment of the genic portion of wheat

To reduce the size and complexity of the wheat genome we used the NimbleGen SeqCap EZ in‐solution custom capture probe set (~110 Mb). The design and targets of the capture probe set that is used here is as previously reported (Gardiner *et al*., 2014). The capture probe set was designed to target 169 345 contigs that varied in size from 100 to 13 168 bp with capture probes of 80–100‐bp tiling across them. The final design was: based on the wheat reference variety Chinese Spring; non‐redundant and covered the majority of the wheat genes; designed so each probe could target three homoeologous gene copies in hexaploid wheat (Gu *et al*., 2004).

### Generating genome references for each parent

Our first step was to construct a set of wheat pseudo‐chromosomes to be used as a mapping reference. These were designed using the set of contiguous sequences that the capture probe set was tiled across (capture design contigs) ordered and anchored onto wheat chromosomes and concatenated.

The initial design for these pseudo‐chromosomes was based on our previously reported set of wheat pseudo‐chromosome sequences that were ordered by aligning wheat sequence to the *Brachypodium* genome to give long‐range order and then using 807 *Brachypodium*–wheat markers to order and associate parts of the *Brachypodium* genome to the corresponding wheat genome (Gardiner *et al*., 2014). Here we improved this approach using 11 016 wheat markers from the MIPS Genome Zipper (Nussbaumer *et al*., 2013; Spannagl *et al*., 2013) that were identified within the International Wheat Genome Sequencing Consortium (IWGSC) wheat Chinese Spring Survey Sequence (CSS) contigs and had been assigned a wheat chromosome cM position (The International Wheat Genome Sequencing Consortium (IWGSC), 2014). BLASTN (version 2.2.17) was used to place the capture design contigs onto the IWGSC CSS contigs (E‐value cutoff 1e‐5). Capture design contigs, hitting an IWGSC contig that contained a marker, could be assigned approximate positional information in wheat. Thus we were able to assign 17 960 of the 169 345 contigs. Furthermore, if these design contigs, now with positional information, aligned to the closely related species *Brachypodium distachyon* then they could be used as *Brachypodium*–wheat markers to estimate positional information for other sequences aligning nearby in *Brachypodium,* as per our previous methodology (Gardiner *et al*., 2014). This resulted in 14 575 design contigs acting as *Brachypodium*–wheat markers. To enable the use of these *Brachypodium*–wheat markers BLASTN was used to place the remaining 151 385 unassigned capture design contigs onto the *Brachypodium* genome. For each contig the top BLAST gene hit was used and hits were prioritized as follows; highest BLAST score, longest length (minimum 100 bp), lowest E‐value (minimum 1e‐3) and highest sequence identity (minimum 90%). The design contigs could then be ordered into wheat pseudo‐chromosomes based on association with the wheat chromosome marker positions.

**Figure 1:**
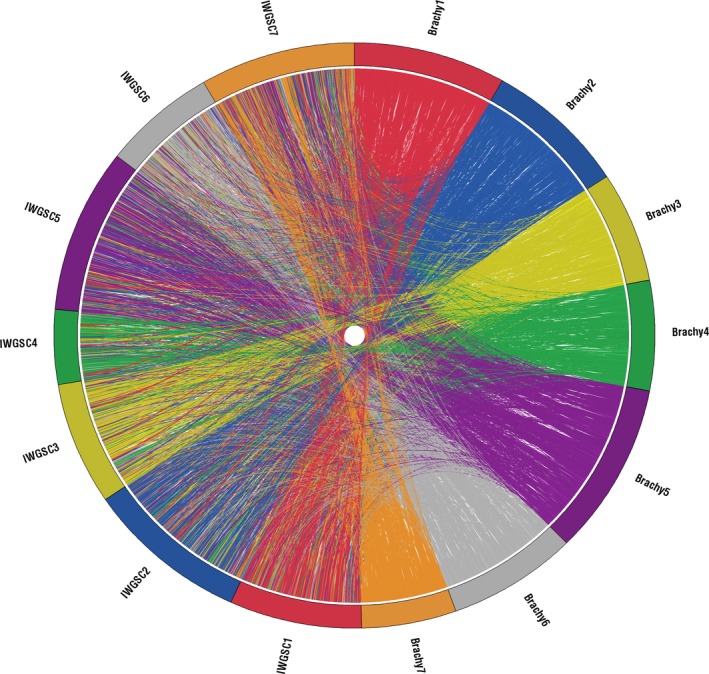
Demonstrating the reorganization of capture probe design contigs between two pseudo‐genome assemblies. Highlighting how contig positions in our previous pseudo‐genome assembly that was generated using 807 *Brachypodium* markers (Gardiner *et al*., 2014), are translated, using connecting ribbons, to their respective positions in the pseudo‐genome that was generated here using 11 016 wheat markers from the Genome Zipper (Nussbaumer *et al*., 2013; Spannagl *et al*., 2013). Chromosomes are labeled according to the markers that were used to generate them; in the previous pseudo‐genome they are labeled Brachy 1–7 and in the newer pseudo‐genome they are labeled IWGSC 1–7. Chromosomes are colour coded and connecting ribbons show the colour of the chromosome of origin of the contig in the previous pseudo‐genome (Brachy 1–7).

This methodology allowed us to associate 118 635 of the original 169 345 capture probe design contigs with a marker and to therefore organize and concatenate them into seven pseudo‐chromosome sequences. A comparison of the capture design contig locations between our previous pseudo‐genome and this updated Genome Zipper‐based pseudo‐chromosome assembly is shown in Figure 1. This Genome Zipper‐based organization is an improvement on the previous set of pseudo‐chromosomes with an additional 3385 contigs included in the assembly and an additional 10 209 markers implemented for more accurate local ordering. This resulted in 47.4% of contigs in the previous pseudo‐chromosome set being assigned a new chromosome with change most prominent for pseudo‐chromosome 4, losing 4453 contigs, and pseudo‐chromosome 7, gaining 7617 contigs. Importantly since the pseudo‐chromosomes are based on the capture design contigs, each pseudo‐chromosome represents the three wheat genomes A, B and D. This results in an overall representation of all 21 wheat chromosomes while homoeologous SNPs can be used downstream for discrimination between them. This pseudo‐genome was used as the reference sequence for mapping analyses within this study to highlight the suitability of this methodology for poorly defined polyploid genomes.

**Figure 2:**
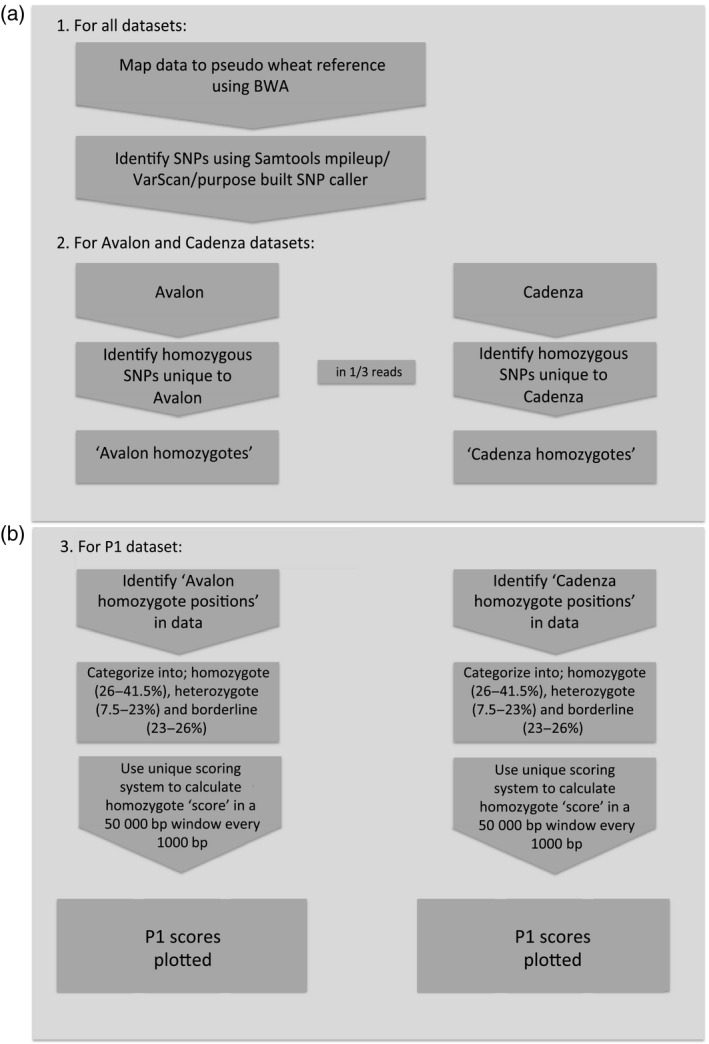
Processing three sets of enriched sequencing data to identify a mapping interval containing the gene that is inducing the phenotype of interest. (a) Standard mapping and SNP calling pipeline to construct ‘reference genomes’. (b) Pipeline implementing an algorithm to score regions of interest by prioritizing long homozygous parental haplotypes for the bulk segregant sample to identify the interval of interest. Default figures shown here of 50 000‐bp windows at 1000‐bp intervals and standard homozygote/heterozygote/borderline SNP definitions shown‐values are adjusted throughout the analysis as necessary.

The pipeline to generate ‘reference genomes’ based on the two purebred parental lines of the varieties Avalon and Cadenza is shown in Figure 2(a). Genomic DNA was isolated from each of the parents and separately enriched using our NimbleGen SeqCap EZ wheat exome capture probe set before sequencing using an Illumina HiSeq 2000. The short read sequences were mapped to the Chinese Spring pseudo‐wheat chromosomes using short read mapping software Burrows‐Wheeler Aligner (BWA) (Li and Durbin, 2009). Coverage of the reference was similar for both the Avalon and Cadenza sequencing datasets. In both experiments approximately 97% of the reference was covered with uniquely mapping, non‐duplicate reads, to an average depth of ~42× (see Table S1).

SNPs were scored between the Chinese Spring reference and the two cultivars using SAMtools mpileup (v0.1.18) (Li *et al*., 2009a) and VarScan (v2.2.11) (Koboldt *et al*., 2012). SNPs with a mapping coverage less than 30× were filtered out [see Table S1]. Parental homoeologous homozygous SNP alleles are expected to account for approximately one‐third of the sequencing reads at a position, as such, they were recorded if the allele was observed in 23–43% of the sequencing reads with the reference allele seen in 57–77% of the reads. We identified 395 162 homoeologous homozygous SNPs in relation to the Chinese Spring reference that were shared between the two parental lines. In addition, we identified homozygous SNPs using Chinese Spring that were specific to Avalon or Cadenza if the homozygous SNP allele was not observed in the other parent at that position resulting in 44 208 Avalon and 70 644 Cadenza specific homozygous SNPs. The parental specific SNPs could then be used to generate Avalon and Cadenza ‘reference genomes’.

### Generation of *Yr6* phenotypic bulks for mapping‐by‐sequencing

Mapping‐by‐sequencing relies on a local skewing of allelic frequency close to the loci of interest, which has a strong enough phenotypic effect to be significantly partitioned when the extreme tails of the phenotypic distribution are separately bulked. Here, we used the Avalon × Cadenza doubled haploid population (see Acknowledgements), whose parents were known to be either susceptible (Avalon) and resistant (Cadenza) to races of yellow rust carrying avirulence to the *Yr6* gene (Pathan *et al*., 2008; Bansept‐Basler, 2013). Our seedling screen of the 201 progeny lines of this population with the UK yellow rust isolate 03/07 avirulent for *Yr6* (AVRYR6) showed a bimodal segregation pattern, with half of the population showing resistance and half clear susceptible reactions (Figure S1), consistent with the segregation of a single major resistance gene, *Yr6*. Ninety‐five individual lines with a score of less than or equal to 2 on a 0–9 scale (corresponding to the complete absence of any sporulation) at 14 days post infection were identified and DNAs from these 95 individuals were combined to create a ‘resistant’ pool referred to henceforth as P1 (Figure 2b). This P1 bulk was enriched using the NimbleGen SeqCap EZ wheat exome capture array in solution and then sequenced. The sequence data that were generated was mapped using BWA, and SNPs scored and filtered as described for the parents, using SAMtools mpileup and VarScan [see Table S1]. A minimum depth of 50× coverage was applied to the SNPs that were called and on average 98% of the reference base space was mapped to uniquely after removal of duplicate reads, at an average depth of ~58× [see Table S1].

### Using an allele frequency determination algorithm to identify a single homozygous peak on chromosome 7

We have previously developed a mapping‐by‐sequencing mutant identification pipeline and algorithm (Gardiner *et al*., 2014). Here, we have adapted and applied the pipeline successfully for the analysis of a polyploid (Figure 2b). Mapping‐by‐sequencing in hexaploid wheat faces unique challenges for confident mapping and SNP calling, particularly due to the presence of three homoeologous sub‐genomes with high similarity. One issue, when implementing gene capture, is that one genome could be preferentially enriched over another. We have previously demonstrated that our capture probes are capable of enriching all three genomes with no significant read bias to one specific sub‐genome (Jordan *et al*., 2015). Also, mapping to the three reference genomes individually results in a high level of non‐uniquely mapped sequencing reads. Furthermore, here the analyzed cultivars are different from the Chinese Spring‐based reference introducing more SNPs and further hindering confident unique mapping. Therefore to maximize mapped sequencing data, and to highlight the suitability of this methodology for poorly defined polyploid genomes where sub‐genome assemblies have not been discriminated, we align reads to a collapsed reference genome rather than three. However, for the application of SNP calling to our hexaploid P1 bulk segregant dataset, a homoeologous SNP allele must be categorized as being homozygous or having heterozygosity within the pool. We defined specific thresholds for this categorization as follows; homoeologous homozygous alleles in 50–24% of sequencing reads; borderline SNP alleles in 23–24% of sequencing reads and homoeologous heterozygosity within a pool is defined if SNP alleles are in 10–23% of sequencing reads (default settings shown in Figure 2b). These thresholds are relatively relaxed to allow the analysis of a maximum number of positions. We then utilize an algorithm to score regions of interest by prioritizing long homozygous parental haplotypes, the longer the length and the more homozygous the region in the pool, the higher the score generated (Gardiner *et al*., 2014).

**Figure 3:**
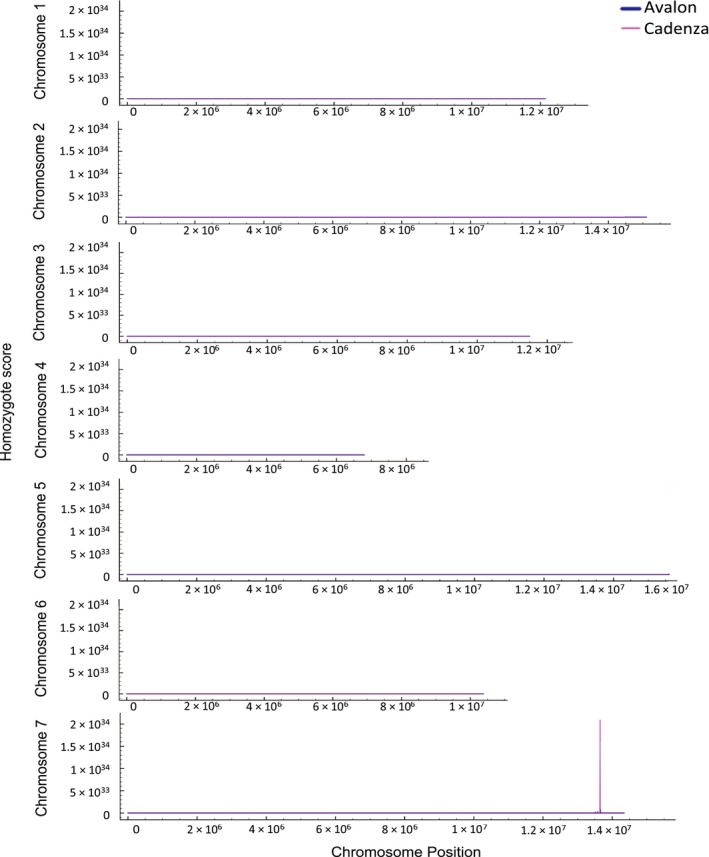
Homozygosity scores calculated for the bulk segregant dataset along each pseudo‐chromosome. Magenta: Scores plotted for ‘Cadenza specific homoeologous homozygote SNP alleles’ found in the bulk segregant dataset. Blue: Scores plotted for ‘Avalon‐specific homoeologous homozygote SNP alleles’ found in the bulk‐segregated dataset. Scores calculated per 500 000‐bp window along each chromosome at 10 000‐bp intervals.

Of the homoeologous homozygote SNPs that were specific to the Avalon parent, 28 260 of the SNP alleles were conserved in the P1 dataset regardless of allele frequency. Similarly of the homozygote SNPs that were specific to the Cadenza parent, 35 639 of these were found in the P1 dataset. The scoring algorithm was then implemented to calculate a homozygosity score per 500 000 bp window along each pseudo‐chromosome at 10 000 bp intervals for SNP alleles that were found in the bulk segregant dataset and were specific to either the Avalon or Cadenza parents (Figure 3).

We identified a single peak towards the end of chromosome 7 (~13.5 Mbp) in Cadenza but not in Avalon (Figure 3). This supports previous studies in which the *Yr6* locus has been reportedly found on wheat chromosome 7B (Li *et al*., 2009b; Ma *et al*., 2015). The defined peak in Figure 3 suggests conserved homozygosity between the bulk segregant mapping population and the Cadenza parental line in this region and is likely to represent the inheritance of the *Yr6* locus from the Cadenza parental line that is thought to confer the shared stripe rust resistance to the AVRYr6 isolate.

The peak interval represented 500 Kbp of genic sequence on pseudo‐chromosome 7 between 13 650 001 and 14 150 001 bp. There were 622 capture probe design contigs, representing gene sequences, that were concatenated to form this region and it encompassed wheat markers within an approximate 21 cM range, from 192.386 to 213.416 cM.

### Candidate region contains potential disease resistance genes and associates with the B sub‐genome on chromosome 7

**Figure 4:**
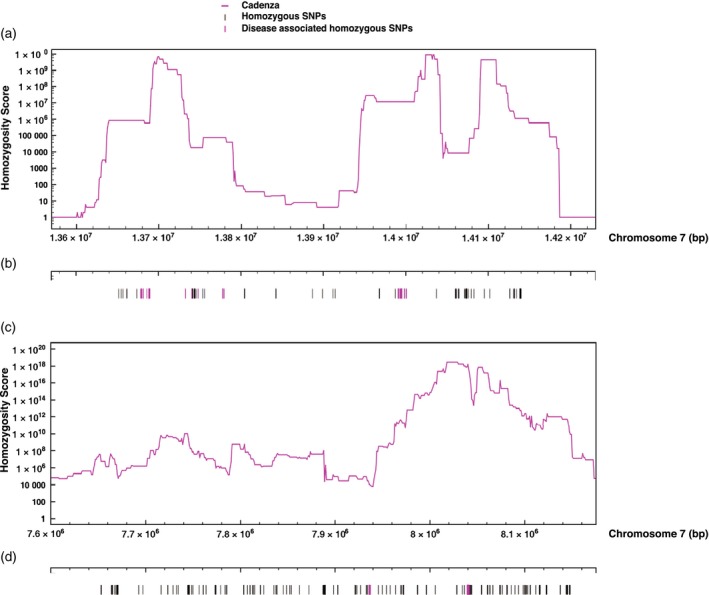
Analysis of the peak intervals on pseudo‐chromosome 7. (a, c) Magenta line: Scores plotted for ‘Cadenza specific homoeologous homozygote SNP alleles’ found in the bulk segregant dataset. Scores calculated per 500 000‐bp window along each chromosome at 10 000‐bp intervals and displayed only for (a) the interval 13 650 001–14 150 001 bp on the MIPS‐derived pseudo‐chromosome 7 and (c) the interval 7 650 001–8 150 001 bp on the POPSEQ‐derived pseudo‐chromosome 7. (b, d) Uses the same *x*‐axis as plots (a, c) respectively to depict the same interval: symbols (|) highlight the positions of candidate SNPs that are highly homozygous in the bulk segregant dataset and conserved with the Cadenza parent, those coloured magenta show SNPs that overlap disease‐resistance‐related genes.

We investigated the interval between 13 650 001 and 14 150 001 bp on pseudo‐chromosome 7 in more detail; however, here a window size of 100 000 bp was used to calculate homozygosity scores to allow us to analyze the interval with greater precision (Figure 4a). We also analyzed this peak interval using a BLASTN alignment (Altschul *et al*., 1990) (minimum E‐value of 1e‐5) to the BLAST nr nucleotide database with a focus on the identification of potential disease resistance genes. It has been previously observed that disease resistance genes in plants commonly encode nucleotide binding site leucine‐rich repeat proteins (NBS‐LRR) therefore the matched sequences are strong candidates for stripe rust resistance (McHale *et al*., 2006). The homologous genes that were identified within the peak interval included six disease‐resistance‐associated genes (Table S2): the first at 13 674 779–13 700 582 and 13 725 771–13 739 999 bp associating with RPM1; the second at 13 745 548–13 749 400 bp associating with RGA3; the third at 13 756 980–13 759 981 bp associating with leaf rust resistance; the fourth at 13 773 200–13 778 547 bp associating with RGA4; the fifth at 13 990 651–13 998 751 bp associating with ENHANCED DISEASE RESISTANCE 2; and finally at 14 000 374–14 003 318 bp associating with an LRR repeat domain‐containing protein. Interestingly, these disease‐resistance‐associated gene regions fall under the main peaks that can be seen in Figure 4(a) allowing us to focus on these smaller candidate intervals amounting to approximately 60 Kbp. The single peak in Figure 3 separates into smaller peaks in Figure 4(a), which is likely to be due to inaccuracies in contig order at a local level.

We also scanned the peak interval between 13 650 001 and 14 150 001 bp on pseudo‐chromosome 7 for candidate homozygous or borderline SNPs. A list of 145 SNPs was identified that were highly homozygous across the pool and conserved between the bulk segregant dataset and the Cadenza parent (Figure 4b and Table S3). In total, 51 of these homozygous SNPs are located within the peaks shown in Figure 4(a) and have disease‐resistance functions (highlighted in Figure 4b and detailed in Table 1).

**Table 1:** Detailing candidate homozygous SNP alleles for stripe rust resistance. Homoeologous homozygous SNPs found in 23–43% of reads in the bulk segregant dataset that are within the peak interval (13 650 001 and 14 150 001 bp) and that could be associated with known disease resistance genes. These SNPs are conserved with unique homozygous SNP alleles, in 23–43% of reads, which were found in the Cadenza parent. Gene annotations if SNP region can be associated with a BLASTN gene hit (E‐value 1e‐5)

Chr	Position	Alternate allele	% reads with alt allele	Amino acid change	Predicted genome of origin	Associated gene
7	13677450	G	40.28	–	–	gi|357127680|ref|XM_003565459.1| *Brachypodium distachyon* disease resistance protein RPM1‐like (LOC100830503) gi|156152300|gb|EF567062.1| *Triticum aestivum* cultivar Glenlea clone BAC 1648_464 disease resistance protein (Lr1) genomic region
7	13677456	A	33.33	–	–	
7	13677457	G	33.85	–	–	
7	13677461	T	36.51	–	–	
7	13677473	T	38.46	–	–	
7	13678233	G	30.00	–	–	
7	13680058	C	32.88	–	–	
7	13680066	A	25.68	–	–	
7	13680112	G	26.83	–	–	
7	13680129	G	25.47	–	–	
7	13684538	C	32.89	–	–	
7	13686827	G	35.19	Phenylalanine → Leucine	–	
7	13686846	G	30.30	Synonymous	–	
7	13686849	C	30.88	Synonymous	–	
7	13686852	C	28.17	Asparagine → Lysine	–	
7	13686867	C	39.73	Synonymous	–	
7	13687809	A	25.93	Synonymous	B	
7	13687869	G	25.00	Synonymous	B	
7	13688052	C	24.00	Synonymous	B	
7	13731758	G	36.54	–	–	
7	13739416	T	32.69	Glutamic acid → Aspartic acid	–	
7	13739467	G	38.46	Synonymous	D	
7	13739566	A	32.35	Synonymous	–	
7	13739662	T	39.34	Synonymous	–	
7	13747104	G	38.10	Synonymous	–	gi|359754734|gb|JN381196.1| *Triticum aestivum* RGA1 (rga1) mRNA, complete cds gi|359754736|gb|JN381197.1| *Triticum aestivum* RGA3 (rga3) mRNA, partial cds gi|11991253|gb|AF320847.1|AF320847 *Triticum aestivum* putative NBS‐LRR disease resistance protein RCCN5 (Rccn5) pseudogene, partial sequence
7	13776813	T	23.86	Arginine → Lysine	–	gi|357150993|ref|XM_003575599.1*| Brachypodium distachyon* putative disease resistance protein RGA4‐like (LOC100832361)
7	13776903	T	23.53	Arginine → Glutamine	–	
7	13778333	G	27.50	Synonymous	–	
7	13990669	T	33.09	Alanine → Serine	–	*Brachypodium distachyon* protein ENHANCED DISEASE RESISTANCE 2‐like (LOC100833403)
7	13990704	T	40.54	Synonymous	–	
7	13991111	C	30.26	Leucine → Proline	–	
7	13991112	A	26.67	Synonymous	–	
7	13991240	A	39.22	Tryptophan → Stop	–	
7	13992689	G	35.29	Isoleucine → Methionine	B	
7	13992691	C	34.62	Cysteine → Serine	B	
7	13992732	C	38.98	Cysteine → Arginine	B	
7	13992873	C	38.46	Tyrosine → Histidine	–	
7	13992902	A	40	Methionine → Isoleucine	–	
7	13992933	G	41.90	Serine → Alanine	–	
7	13993115	C	38.10	Synonymous	B	
7	13993116	A	37.65	Glycine → Arginine	B	
7	13993129	G	28.21	Glutamine → Arginine	B	
7	13994091	A	32	Phenylalanine → Tyrosine	B	
7	13994429	C	26.37	Tyrosine → Histidine	–	
7	13994435	C	26.67	Tyrosine → Histidine	–	
7	13995096	T	24	Alanine → Valine	D	
7	13995120	C	33.33	Leucine → Proline	–	
7	13995144	T	27.27	Alanine → Valine	–	
7	13998180	A	41.86	Proline → Leucine	B	
7	13998354	C	33.33	Tyrosine → Cysteine	B	
7	14000698	A	23.08	–	–	gi|357146449|ref|XM_003573948.1| *Brachypodium distachyon* LRR repeats and ubiquitin‐like domain‐containing protein At2g30105‐like (LOC100821123)

The SNPs detailed in Table 1 were defined as synonymous/non‐synonymous whenever possible. Their surrounding sequence (±500 bp) was used in a BLASTX alignment of the translated nucleotide query to the BLAST nr protein database (Altschul *et al*., 1990) (minimum E‐value 1e‐5). If the reading frame for translation was conserved across the top three hits to disease‐resistance genes (longest length and lowest E‐values) then this reading frame was used to determine downstream SNP effect. 24 of the SNPs were predicted as non‐synonymous using this methodology. Furthermore, 100 bp sequencing reads containing the specific SNP alleles were extracted and used in a BLASTN alignment (minimum E‐value of 1e‐5) against the IWGSC CSS chromosome 7 contigs. If the top scoring read alignment was to a single contig with 100% sequence identity then an alignment was confidently recorded. This allowed us to predict the genome of origin of the SNP allele (Table 1). Of the SNPs that could be genome‐associated ~85.7% were found on chromosome B with ~14.3% on chromosome D. This highlights the likelihood of the defined interval being associated with genome B on chromosome 7.

### Using POPSEQ‐derived pseudomolecules to re‐define our pseudo‐chromosome reference sequences

Rather than implement the markers that were defined by the wheat Genome Zipper to order the capture design contigs, we made use of 21 wheat chromosomal pseudomolecules that were created by organizing and concatenating the IWGSC CSS assemblies using POPSEQ data (Chapman *et al*., 2015). BLASTN was used to place the capture design contigs onto these chromosomal pseudomolecules (E‐value cutoff 1e‐5, minimum sequence identity 90% and minimum length of 100 bp). In total, 96 781, 116 220 and 97 329 capture design contigs hit the genome A, B and D chromosomal pseudomolecules respectively. Relative positions for the capture design contigs along the chromosomal pseudomolecules could then be used to order them into our POPSEQ‐based pseudo‐chromosomes. We required seven POPSEQ‐based pseudo‐chromosomes, as per our capture probe set, that were representative of the 21 wheat chromosomes. Therefore the order of the capture design contigs along genome B's chromosomal pseudomolecules 1–7 was preferentially utilized as the greatest number of contigs could be aligned to these sequences.

**Figure 5:**
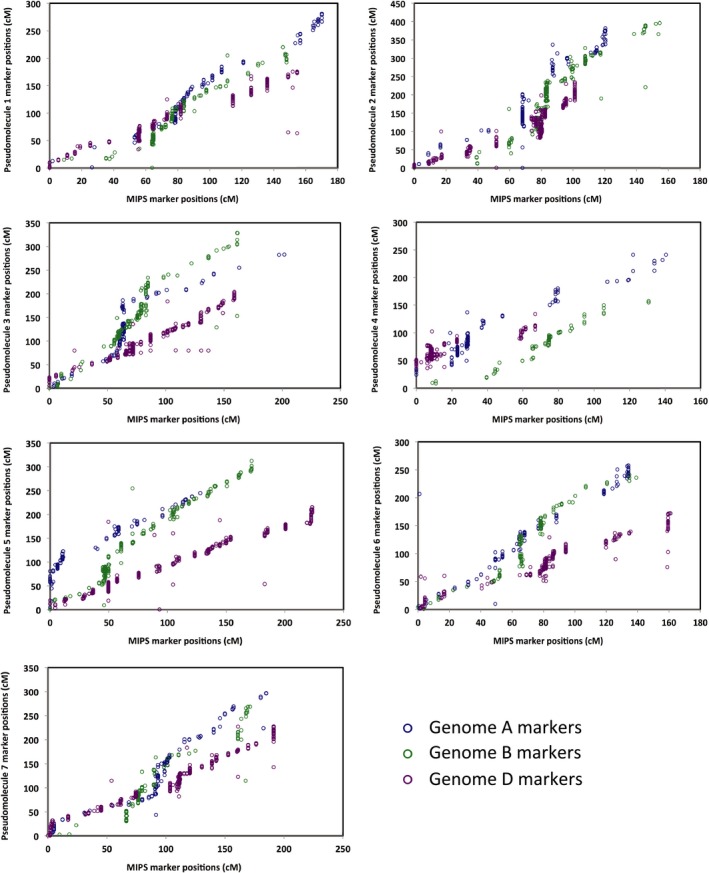
POPSEQ versus Genome Zipper genetic markers. Detailing 6043 IWGSC CSS contigs that have been assigned a genetic position (cM) by the Genome Zipper and also in the POPSEQ analysis. Contigs included only if both the Genome Zipper and POPSEQ analyses assign them to the same sub‐genome and chromosome.

This POPSEQ pseudo‐genome contained 116 220 of the 169 345 capture design contigs; 2415 less than the previous Genome Zipper‐based design though 970 more than our previously published assembly (Gardiner *et al*., 2014). POPSEQ genetic positions are available for the IWGSC CSS contigs that aided their assembly into chromosomal pseudomolecules. Here, 6043 of these IWGSC CSS contigs also had Genome Zipper assigned genetic positions and these positions are compared in Figure 5.

Figure 5 shows, as anticipated, a positive correlation for contig genetic positions between the Genome Zipper and POPSEQ datasets that is conserved across the seven chromosomes. However, data points are more dispersed, demonstrating some discongruity between the marker sets.

The physical positions of the capture design contigs within the genome B chromosomal pseudomolecules were compared with their Genome Zipper assigned genetic positions in the A, B or D genome (Figure S2). The characteristic sigmoid shape seen in each of the plots in Figure S2 differs from the positive correlation seen in Figure 5. This highlights the likely reduced recombination activity towards centromeric regions, resulting in greater physical distances represented by the same cM distance. Furthermore a high degree of conservation can be observed between the A, B and D genome Genome Zipper marker positions and their physical positions within the genome B chromosomal pseudomolecules via their association with the capture design contigs. This demonstrates a successful integration of the three genomes into one representative genome per chromosome.

### Mapping‐by‐sequencing of the *Yr6* locus using POPSEQ‐derived pseudo‐chromosome reference sequences

The mapping‐by‐sequencing analysis of the *Yr6* locus was repeated using the re‐designed POPSEQ‐based pseudo‐chromosome reference sequence. The same parameters for the mapping and SNP calling pipeline were used as in the previous analysis. Across the three sequencing datasets (Avalon, Cadenza and the bulk), coverage was highly conserved with on average 98% of the reference sequence being mapped to uniquely after removal of duplicate reads. An average depth of coverage of 45× was seen in the parental lines and 60× in the P1 bulk (Table S1)**.** We identified 37 322 Avalon‐specific and 58 868 Cadenza‐specific homozygous SNPs.

Of the homoeologous homozygote SNPs that were specific to the Avalon parent, 23 767 of the SNP alleles were conserved in the bulk segregant dataset regardless of homozygous or heterozygous status. Similarly, of the homozygote SNPs that were specific to the Cadenza parent, 29 608 of these were found in the bulk segregant dataset. These SNPs were categorized as homozygous, heterozygous or borderline according to the hexaploid‐specific thresholds used previously and the scoring algorithm was implemented to calculate a homozygote score per 500 000 bp window along each pseudo‐chromosome at 10 000 bp intervals (Figure S3).

Using our POPSEQ‐based pseudo‐chromosome reference we observed a single peak near the end of pseudo‐chromosome 7 in Cadenza but not in Avalon at 7 650 001–8 150 001 bp (500 Kbp of genic sequence) (Figure S3). Using the BLAST alignment of the contigs to the POPSEQ pseudomolecules that was used for ordering, the interval could be translated to ~239 000 001–245 000 000 bp on the POPSEQ chromosomal pseudomolecule 7B and to POPSEQ markers 148.22–167.91 cM. Interestingly, the central point of this region overlaps previous estimates of the *Yr6* locus using the *gwm577* marker that is reported at 157 cM on Chinese Spring chromosome 7B.

### Using the POPSEQ‐derived pseudo‐genome, the defined region on chromosome 7 overlaps the Genome Zipper‐based result

The defined interval using the POPSEQ‐based pseudo‐genome (148.22–167.91 cM) highlights discrepancies between the POPSEQ and Genome Zipper marker sets since it differs from the region that was identified at 192.386–213.416 cM on chromosome 7 using the Genome Zipper‐based pseudo‐genome. However, if we look at the IWGSC CSS contigs in the 239 000 000–245 000 000 bp interval on the POPSEQ chromosomal pseudomolecule 7B, 16 contigs also had a Genome Zipper cM position that translated to 199.94–265.29 cM on chromosome 7 and therefore overlaps the previous region (192.386–213.416 cM).

The interval 7 650 001–8 150 001 bp on the POPSEQ‐based pseudo‐chromosome 7 contained 709 capture design contigs, or gene sequences, that were concatenated to form this region. 29.5% of these contigs were not included in the Genome Zipper‐based pseudo‐chromosomes and similarly 35.4% of the contigs within the Genome Zipper peak interval of 13 650 001–14 150 001 bp were not included in the POPSEQ‐based pseudo‐chromosomes; this highlights the necessity of these complementary approaches for full annotation of the locus. However, looking at the contigs within the 7 650 001–8 150 001 bp interval on the POPSEQ‐based pseudo‐chromosome 7 that were conserved with the Genome Zipper pseudo‐chromosome 7, 82% were found within 1 Mbp of the Genome Zipper peak interval of 13 650 001–14 150 001 bp and, furthermore, 60% of these contigs were found within the peak interval itself. Therefore there is significant crossover between the peak intervals.

We analyzed the POPSEQ‐based interval of interest with greater precision by using a window size of 100 000 bp to calculate homozygosity scores (Figure 4c). We also analyzed this peak interval using a BLASTN alignment (minimum E‐value of 1e‐5) to the BLAST nr nucleotide database with a focus on the identification of potential disease resistance genes (Altschul *et al*., 1990). Homologous genes identified within the peak interval included three disease resistance associated genes (Table S4): the first at 7 885 679–7 887 186 bp associating with putative disease resistance; the second at 7 936 847–7 938 817 bp also associating with putative disease resistance; and finally at 8 040 733–8 043, 356 bp associating with a NBS‐LRR protein. Two of these disease‐resistance‐associated gene regions fall in the immediate vicinity of the main peak that can be seen in Figure 4(c), allowing us to focus on these smaller candidate intervals amounting to around 6 Kbp.

We also scanned the peak interval for candidate homozygous or borderline SNPs. A list of 239 SNPs was identified that were highly homozygous across the pool and conserved between the bulk segregant dataset and the Cadenza parent (267 identified in total, 28 conserved with previous list in Table S2 and 68 across chromosome 7) (Figure 4d and Table S5). 18 of these homozygous SNPs were associated with the gene regions that showed disease resistance functions (highlighted in Figure 4d and detailed in Table 2). These SNPs were defined as synonymous/non‐synonymous where possible as per previous methodologies, resulting in seven SNPs that were predicted as non‐synonymous. Genomes of origin were predicted for the SNPs using previous methodologies; 94.4% of SNPs were associated with genome B and 5.6% were associated with genome D.

**Table 2:** Detailing candidate homozygous SNP alleles for stripe rust resistance (defined using a POPSEQ‐based pseudo‐genome). Homoeologous homozygous SNPs found in 23–43% of reads in the bulk segregant dataset that are within the peak interval (7 650 001 and 8 150 001 bp) and that could be associated with known disease resistance genes. These SNPs are conserved with unique homozygous SNP alleles, in 23–43% of reads, which were found in the Cadenza parent. Gene annotations if SNP region can be associated with a BLASTN gene hit (E‐value 1e‐5)

Chr	Position	Alternate allele	% reads with alt allele	Amino acid change	Predicted genome of origin	Associated gene
7	7936900	G	32.0	Leucine → Valine	B	gi|575868673|gb|KF810140.1| *Triticum aestivum* NBS‐LRR protein (ScRGA‐6RL1) gene, complete cds *Avena strigosa* clone L7M2.3 putative resistance protein gene, partial cds
7	7937040	T	28.9	Synonymous	B	
7	7937151	G	26.1	Synonymous	B	
7	7937157	A	23.0	Synonymous	B	
7	7938413	A	29.5	–	B	
7	8040766	T	36.3	Synonymous	B	gi|82527212|gb|DQ256077.1| *Triticum aestivum* clone Tp3a5a powdery mildew resistance protein pseudogene, partial sequence gi|244536905|emb|FM876846.1| *Thinopyrum ponticum* ag15 (NBS‐LRR) gene, 7AgL allele, exons 1–2
7	8040868	T	33.3	Synonymous	B	
7	8040954	C	34.4	Isoleucine → Threonine	B	
7	8040970	T	32.5	Synonymous	B	
7	8041152	C	30.3	Synonymous	B	
7	8041610	G	30.5	Aspartic Acid → Glycine	B	
7	8041655	T	31.3	Alanine → Valine	B	
7	8041794	A	35.2	Alanine → Threonine	B	
7	8042015	C	28.0	Synonymous	B	
7	8042027	A	33.3	Synonymous	B	
7	8042311	T	23.08	Threonine → Isoleucine	D	
7	8042917	A	29.31	Synonymous	B	
7	8042925	C	24.53	Serine → Threonine	B	

The 18 candidate SNP locations that were defined using the POPSEQ‐based pseudo‐chromosomes (Table 2) were associated with 11.7 cM on pseudo‐chromosome 1 in the Genome Zipper pseudo‐genome and as such had not been previously considered as candidates. Furthermore, of the 51 candidate SNPs that were identified using the Genome Zipper pseudo‐genome (Table 1) 98% were in contigs that were not included in the POPSEQ pseudo‐chromosome 7, and the remaining 2% overlapped within 3 Kbp of the 7 650 001–8 150 001 bp interval. This again highlights the complementarity of these approaches for association of SNPs with the interval.

Finally, we scanned the peak interval between 13 650 001 and 14 150 001 bp on the Genome Zipper pseudo‐chromosome and the interval between 7 650 001 and 8 150 001 bp on the POPSEQ‐based pseudo‐chromosome for candidate homozygous or borderline SNPs that were conserved between the bulk segregant dataset and the Avalon parent. A combined list of 112 SNPs was identified that were highly homozygous across the pool and, as per previous methodologies, their genome of origin was predicted. Of the SNPs that could be genome‐associated 75% were found on chromosome A with 18.3% on chromosome D and only 6.7% on chromosome B. This tendency for A and D genome linked SNPs fits our expectation; since the mutant interval was identified in this region on genome B that was inherited from the Cadenza parent and therefore we would expect the remaining SNP alleles in the interval that are linked to Avalon to be affiliated with genome A or D only.

### Mapping‐by‐sequencing of the *Yr6* locus using the POPSEQ chromosomal pseudomolecules directly to validate our pseudo‐genomes

In wheat, unlike many other complex polyploids, although the reference sequence is incomplete, it is constantly being improved and currently allows distinction between the sub‐genomes A, B and D. Therefore the current POPSEQ wheat chromosomal pseudomolecules provide a likely adequate reference for mapping‐by‐sequencing analyses (Chapman *et al*., 2015). As such, the mapping‐by‐sequencing analysis of the *Yr6* locus was repeated using these chromosomal pseudomolecules directly as a mapping reference to validate our previous analyses using the pseudo‐chromosomes.

In this analysis the same parameters for mapping were used as in the previous analyses. For the sequencing datasets Avalon, Cadenza and P1 29, 27 and 41% of the reference sequence respectively was mapped to uniquely after removal of duplicate reads. Coverage is seen to extend beyond the 110 Mb capture space due to the use of paired end sequencing reads and the presence of off target carryover sequence. An average depth of coverage of 5× was seen in the parental lines and 6× in the bulk (Table S1)**.** This, as anticipated demonstrates a marked decrease in depth of coverage compared to the pseudo‐chromosomes due to the inclusion of low coverage off target sequence data, the separation of the sub‐genomes and additional mapping losses from non‐uniquely mapped reads. As such, SNPs were called as previously but with a minimum depth of 10 for the parental lines and 20 in the P1 dataset. We identified 211 307 Avalon and 237 577 Cadenza specific homozygous SNPs.

Of the homoeologous homozygote SNPs that were specific to the Avalon parent, 147 756 of the SNP alleles were conserved in the bulk segregant dataset regardless of homozygous or heterozygous status. Similarly of the homozygote SNPs that were specific to the Cadenza parent, 143 462 of these were found in the bulk segregant dataset. The sub‐genome discrimination in the wheat reference sequence renders hexaploid wheat effectively as a diploid genome, therefore these SNPs were categorized as homozygous, heterozygous or borderline according to the diploid‐specific thresholds used in our previous study i.e. homozygote in 80–100%, heterozygote in 30–70% and borderline in 71–79% of reads (Gardiner *et al*., 2014) and the scoring algorithm was implemented to calculate a homozygote score per 500 000 bp window along each pseudo‐chromosome at 10 000 bp intervals (Figure S4).

We identified a single peak that extended from ~239 000 001 to 244 450 001 bp with a plateau of 510 Kbp from 239 680 001 to 240 190 001 bp on chromosome 7B in Cadenza but not in Avalon (Figure S4). This peak interval fully overlaps the interval that was defined on chromosome 7B using the POPSEQ‐derived collapsed pseudo‐chromosome reference acting as a validation for this methodology.

## Discussion

We have studied a yellow rust resistant bulk segregant doubled haploid population that was developed from an F1 cross of parental purebred hexaploid wheat lines. We have performed target enrichment for genic regions and, using mapping‐by‐synteny, identified a genic region of 500 Kbp and further narrowed this down to ~60 Kbp on chromosome 7B that is likely to be linked to the *Yr6* stripe rust resistance locus.

It has been noted previously that, at a minimum of 15× coverage, larger pools of ~200 plants generated interval sizes 159–603 Kbp, while smaller pools of ~50 plants generated interval sizes 216–1350 Kbp (James *et al*., 2013). Here the pool size was 95 therefore our initial interval of 500 Kbp is within the anticipated range and our final interval of ~60 Kbp exceeds expectation.

This study extends a proof of concept approach combining genic enrichment and a sliding window mapping‐by‐synteny analysis using a pseudo‐genome that was carried out in the diploid wheat species *T. monococcum* (Gardiner *et al*., 2014). This previous analysis identified a region on chromosome 3 that was likely to contain the *Eps‐3A* ^*m*^ deletion that results in early flowering. The combination of sliding window analyses and mapping‐by‐synteny, implementing a pseudo‐genome directly in a hexaploid, pushes this methodology further. Not only do we successfully apply this approach to wheat to define an interval using the effectively diploid mapping reference of the POPSEQ chromosomal pseudomolecules (Chapman *et al*., 2015), we can also locate the same interval with a fully polyploid wheat reference i.e. our two new and improved pseudo‐genome assemblies that were developed from 11 016 MIPS Genome Zipper wheat markers and also POPSEQ generated markers plus derived chromosomal pseudomolecules (Chapman *et al*., 2015). In implementing these fine‐tuned pseudo‐genomes, we have defined in both cases a relatively small and similar sized genic interval of 500 Kbp on chromosome 7. The collapsed pseudo‐chromosomes represent ~80% of the 110 MB capture space that is thought to represent the majority of wheat genes. These pseudo‐chromosomes allow us to demonstrate that this methodology could be applied to even poorly defined polyploid genomes, such as the autopolyploid sugarcane genome or the allopolyploid cultivated strawberry genome (*Fragaria* × *ananassa*), where we may have incomplete chromosomes, approximate ordering of sequence, gene sequence only (full or partial) or collapsed sub‐genome assemblies.

At first sight, the intervals that were defined using the two pseudo‐genomes differed (POPSEQ‐based versus MIPS Genome Zipper‐based) and there is a degree of discongruity between the marker sets. However, if we use one set of markers to compare the intervals we can clearly identify an overlapping region. Using the Genome Zipper marker set this overlap translates to 199.94–213.42 cM on chromosome 7 i.e. ~13.5 cM. Moreover, looking at the contigs within the 7 650 001–8 150 001 bp interval on the POPSEQ‐based pseudo‐chromosome 7 that were conserved with the Genome Zipper pseudo‐chromosome 7, 82% were found within 1 Mbp of the Genome Zipper peak interval of 13 650 001–14 150 001 bp. While overlapping SNPs and contigs were defined across the intervals that were defined by the two pseudo‐genomes, acting as a validation of the methodology, they also each contributed additional candidate SNPs for further analysis that the other did not, highlighting the benefit of the use of both marker sets and the need in future for further integration of wheat resources. As such, the pseudo‐chromosome reference sequences will be under constant development as resources advance.

In total, 384 homozygous SNP positions that were conserved with the Cadenza parent were identified across the defined intervals from both pseudo‐genome analyses. Furthermore 69 of these SNPs were located in genes with homology to disease resistance NBS‐LRR genes, with 31 predicted to be non‐synonymous. These non‐synonymous SNPs are therefore strong causal candidates for the *Yr6* locus for yellow rust resistance (McHale *et al*., 2006). Across all of the SNPs that could be genome‐associated, 90.6% were found on chromosome B therefore the defined interval was associated with genome B on chromosome 7, similarly to the analysis using the diploid POPSEQ chromosomal pseudomolecules (Chapman *et al*., 2015) and as previously reported (Li *et al*., 2009b). The small candidate gene regions that are identified by this methodology can be annotated using further comparative analyses and gene targets can be, for example, knocked out to confirm functionality.

We have demonstrated the use of a target enrichment strategy using capture probes that have been designed against the hexaploid wheat Chinese Spring to enrich the genic portion of a closely related hexaploid wheat variety gaining up to 60× mapping coverage across 98% of the collapsed pseudo‐chromosome reference sequence. Furthermore, previously we were able to use a collapsed Chinese Spring capture set and reference to analyze *Triticum monococcum,* that is closely related to the bread wheat genome A progenitor (Gardiner *et al*., 2014), suggesting that it may be possible to use a diploid relative as a collapsed reference for mapping and capture in a polyploid using this mapping‐by‐sequencing methodology.

This analysis has taken the principles that we demonstrated previously in diploid wheat (Gardiner *et al*., 2014) and applied them to polyploid wheat using a unique homozygote‐scoring algorithm that highlights longer homozygous haplotypes shared between the mutant parental line and the bulk segregant mutant dataset. This algorithm was implemented to identify and associate SNPs with the *Yr6* locus that is thought to cause stripe rust resistance. The current pipeline is available as a workflow for public use within the iPlant collaborative web portal (see Experimental procedures and Figure S5 for sample output) (The iPlant Collaborative, 2011). Simple adjustment of the definitions of a homozygous, heterozygous and borderline SNP within the analysis will allow it to be easily adaptable to other polyploid species, particularly those with closely related genomes. Application of this methodology to bread wheat demonstrates a rapid method to identify markers and potentially genes underlying key phenotypic traits. In addition, the two pseudo‐genome reference sequences are available for community use within the iPlant collaborative web portal, where we predict that they will become a valuable resource for mapping‐by‐sequencing analyses.

## Experimental procedures

### Phenotyping of the Avalon × Cadenza doubled haploid population for reaction to an AVRYr6 YR isolate

The wheat yellow rust isolate ‘07/03’ is referred to by (Ma *et al*., 2015) as ‘03/07’ and also referred to in the literature as the ‘Brock’ isolate. This isolate was obtained from the UK Cereal Pathogen Virulence Survey collection at NIAB (Cambridge) and was used in a replicated inoculated seedling test of Avalon (susceptible to ‘07/03’) and Cadenza (resistant to ‘07/03’) parental lines and 201 doubled haploid progeny. Rust symptoms were scored 14 days after inoculation on the 0–9 scale described by McNeal *et al*. (1971) in which a score of 3 or less is considered highly resistant.

### Sequence capture and sequencing protocol for the Avalon, Cadenza and bulk segregant datasets

Genomic DNA was extracted from parental cultivars Avalon and Cadenza along with the bulk segregant sample using the Qiagen DNeasy plant mini kit; 2 μg of genomic DNA for each sample, in a total volume of 130 μl, was sheared for 3 × 60 sec using a Covaris S2 focused ultrasonicator (duty cycle 10%, intensity 5, 200 cycles per burst using frequency sweeping). Fragmented DNA was then purified using 1.8× Agencourt AMPure XP beads (Beckman Coulter). The size distribution of the fragmented DNA was assessed on a Bioanalyser high sensitivity DNA chip (Agilent) and the concentration determined using a Qubit double‐stranded DNA high sensitivity assay kit and Qubit fluorometer (Life Technologies); 1 μg of the fragmented purified DNA was used as input for library preparation. All steps, including pre‐capture library synthesis, hybridisation to custom wheat NimbleGen sequence capture probes, and washing, recovery and amplification of captured DNA were carried out according to the NimbleGen SeqCap EZ SR User's Guide (Version 4.0, January 2013) with a couple of small modifications. As the input DNA was derived from wheat, 10 μl of Plant Capture Enhancer (NimbleGen) was used in the hybridisation step instead of Cot human DNA. Also, for the final post‐capture PCR, 16 cycles were used instead of the usual 18.

Final libraries were quantified by Qubit double‐stranded DNA high sensitivity assay and the size distribution ascertained on a Bioanalyser high sensitivity DNA chip. Libraries for the parental cultivars Avalon and Cadenza were pooled in equimolar amounts based on the aforementioned Qubit and Bioanalyser data. This pool and the library for the bulk segregant sample were quantified by qPCR, using an Illumina Library Quantification Kit (KAPA, Biosciences, London, UK) on a Roche LightCycler 480 II system.

Sequencing was carried out on two lanes of an Illumina HiSeq 2000, using version 3 chemistry, generating 2 × 100‐bp paired‐end reads (the parental cultivars on one lane and the bulk segregant sample on the other lane).

#### Mapping and SNP identification in the Avalon, Cadenza and bulk segregant datasets (Figure 2a)

The sequence data was mapped to both of the pseudo‐chromosome reference sequences individually using the short read mapping software BWA's (v 0.7.10; Li and Durbin, 2009) bwa‐MEM algorithm. Indexing of the reference sequence involved use of the ‘IS’ algorithm. All unmapped, non‐uniquely mapped/low quality (MAPQ < 10) and duplicate reads were removed from the analysis. SAMtools mpileup (v 0.1.18) (Li *et al*., 2009a) was implemented on the four datasets and SNP calls were filtered out using VarScan (v2.3.7) (Koboldt *et al*., 2012) with the following parameters: discard SNPs covered by 20 or fewer reads, discard sequencing reads with a quality less than 15 and if the alternate allele has less than two supporting reads passing the quality filter discard it. For this SNP analysis indels were removed from the VarScan output.

#### Implementing the mapping, SNP calling and homozygote haplotyping algorithm in iPlant

Our mapping, SNP calling and haplotyping pipelines are available on iPlant as a workflow within the Discovery Environment; ‘Hexaploid Wheat Mapping By Sequencing Haplotyper’. This workflow requires a mutant parental line, a wild‐type parental line and a bulk segregant mutant pool as input, as was used within this study. Users will also require one of two pseudo‐chromosome wheat reference genomes, along with their corresponding files containing chromosome length, these are also available publicly on the iPlant datastore:/iplant/home/shared/iplant_uk_training/mapping_by_seq_hexaploid‐wheat. To use the workflow with the default settings used in this study, users can simply enter the sequence data files, reference sequence and chromosome length file as prompted in the app followed by clicking launch analysis. The authors recommend using the default parameters, however many can be altered if required. An example of the pdf output generated by iPlant showing the mapping interval for the mutant pool using the Cadenza specific homozygote list is outlined in Figure S5. The workflow also retains all intermediate files from mapping, SNP calling and haplotyping analysis stages. The text file of homozygosity scores used to plot this is also generated as output.

## Supporting information


**Figure S1.** Seedling screen of 201 progeny lines of a doubled haploid population.
**Figure S2.** Physical positions and genetic marker positions of capture design contigs.
**Figure S3.** Homozygosity scores calculated for the bulk segregant dataset along each POPSEQ‐based pseudo‐chromosome.
**Figure S4.** Homozygosity scores calculated for the bulk segregant dataset along each POPSEQ chromosomal pseudomolecule.
**Figure S5.** Homozygosity scores calculated and plotted using workflows implemented through iPlant for the bulk segregant dataset along the MIPS‐based pseudo‐chromosomes.

**Table S1.** Summary of mapping statistics across the pseudo‐chromosome reference
**Table S2.** Detailing gene regions within the peak interval 13 650 001–14 150 001 bp on the Genome Zipper‐based pseudo‐chromosome 7
**Table S3.** Detailing homozygous SNP alleles, in 23–43% of reads, in the bulk segregant dataset within the peak interval (13 650 001 and 14 150 001 bp)
**Table S4.** Detailing gene regions within the peak interval 7 650 001–8 150 001 bp on the MIPS‐based pseudo‐chromosome 7
**Table S5.** Detailing homozygous SNP alleles, in 23–43% of reads, in the bulk segregant dataset within the peak interval (7 650 001 and 8 150 001 bp)
