# Supplementary material for: Mapping‐by‐sequencing in complex polyploid genomes using genic sequence capture: a case study to map yellow rust resistance in hexaploid wheat
Source: Plant J. 2016 Jul 18;87(4):403–19. doi: 10.1111/tpj.13204 (PMC5026171; doi:10.1111/tpj.13204)
Supplement: Supplementary file 1 — Figure S1. Seedling screen of 201 progeny lines of a doubled haploid population. Figure S2. Physical positions and genetic marker positions of capture design contigs. Figure S3. Homozygosity scores calculated for the bulk segregant dataset along each POPSEQ‐based pseudo‐chromosome. Figure S4. Homozygosity scores calculated for the bulk segregant dataset along each POPSEQ chromosomal pseudomolecule. Figure S5. Homozygosity scores calculated and plotted using workflows implemented through iPlant for the bulk segregant dataset along the MIPS‐based pseudo‐chromosomes. [file TPJ-87-403-s001.docx]

**SUPPLEMENTARY DATA**

**
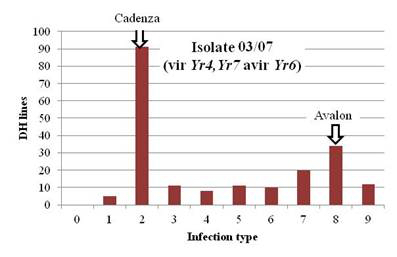
**

**Figure S1. Seedling screen of 201 progeny lines of a doubled haploid population.** Histogram to show the number of doubled haploid lines with each infection type on a scale of 0-9. Infection tending towards 0 corresponds to a complete absence of sporulation. Highlighted are the respective phenotypes of Avalon and Cadenza parental cultivars.


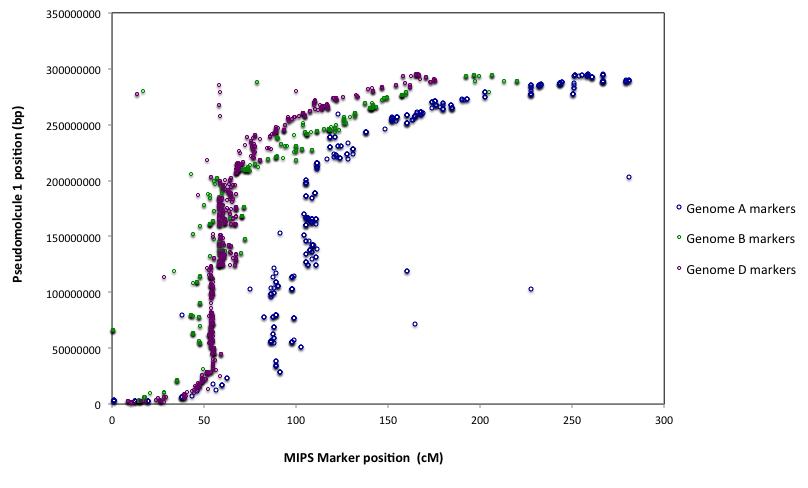

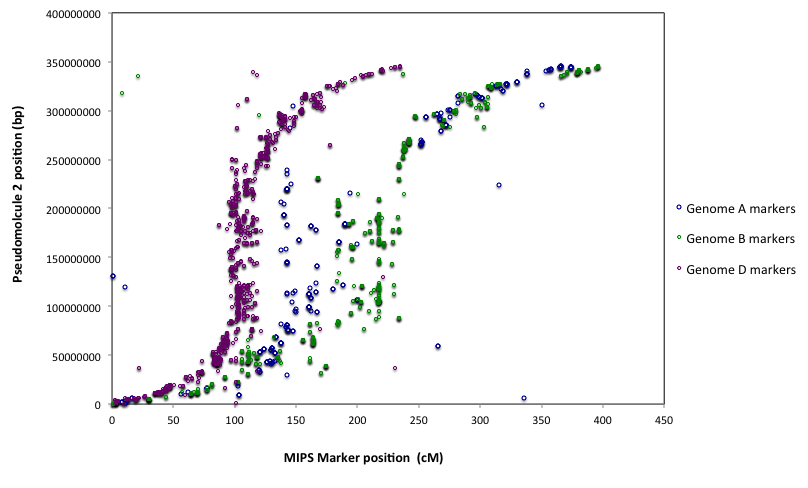


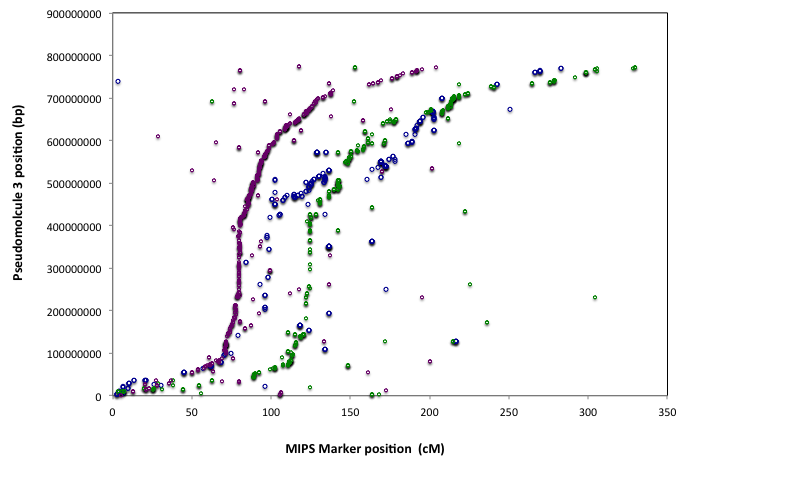

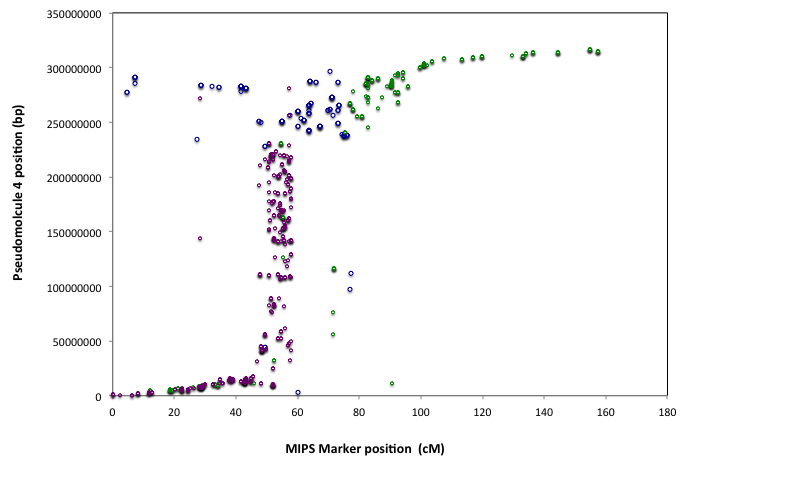


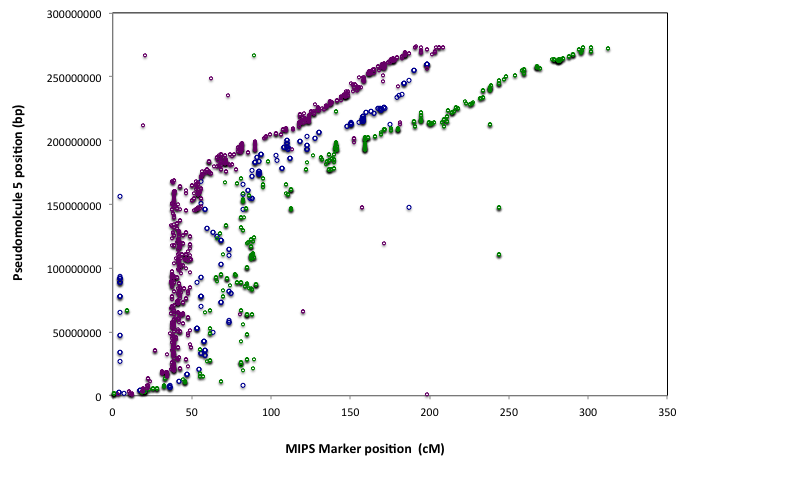

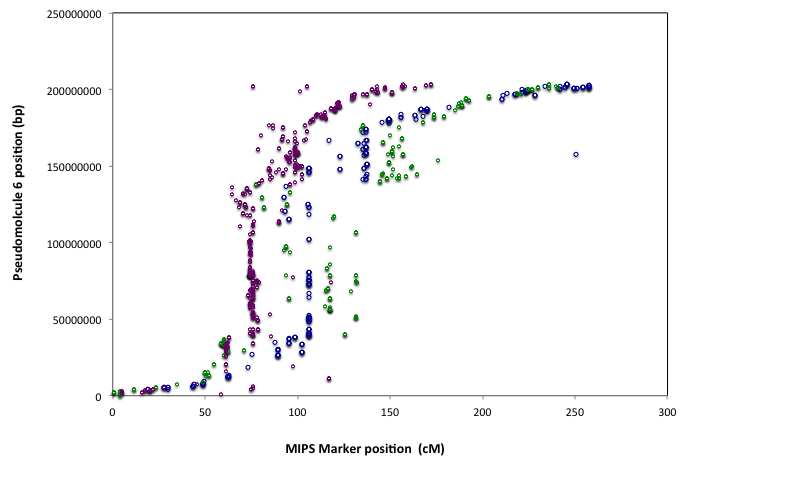


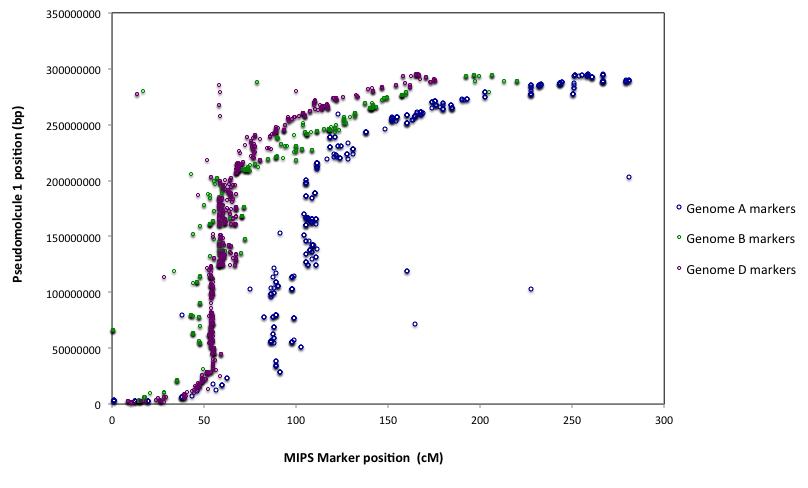

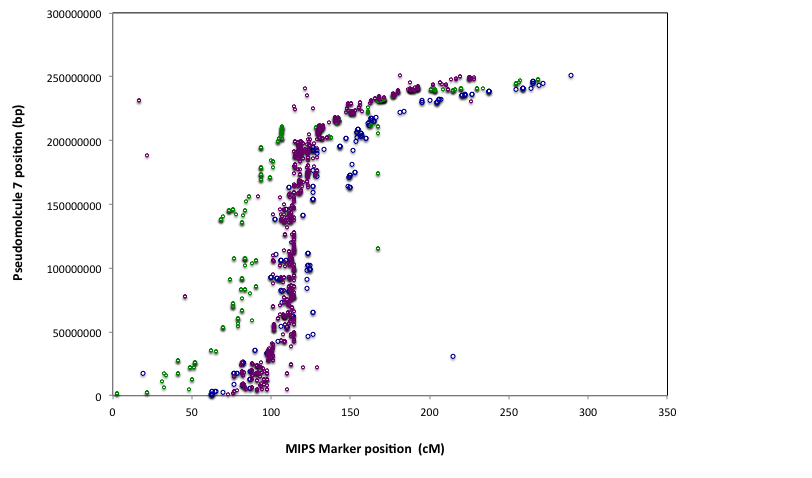


**Figure S2. Physical positions and genetic marker positions of capture design contigs.** Comparing the physical positions (bp) of 12,900 capture design contigs within the genome B chromosomal pseudomolecules, to their Genome Zipper assigned genetic positions in the A, B and D genomes (cM).

**Figure S3.** **Homozygosity scores calculated for the bulk segregant dataset along each POPSEQ based pseudo-chromosome.** Magenta; Scores plotted for ‘Cadenza specific homoeologous homozygote SNPs’ found in the bulk segregant dataset. Blue; Scores plotted for ‘Avalon specific homoeologous homozygote SNPs’ found in the bulk-segregated dataset. Scores calculated per 500,000bp window along each chromosome at 10,000bp intervals.

Chromosome Position (bp)

**Figure S4.** **Homozygosity scores calculated for the bulk segregant dataset along each POPSEQ chromosomal pseudomolecule.** Magenta; Scores plotted for ‘Cadenza specific homoeologous homozygote SNPs’ found in the bulk segregant dataset. Blue; Scores plotted for ‘Avalon specific homoeologous homozygote SNPs’ found in the bulk-segregated dataset. Scores calculated per 500,000bp window along each chromosome at 10,000bp intervals.

**Figure S5.** **Homozygosity scores calculated and plotted using workflows implemented through iPlant for the bulk segregant dataset along the MIPs based pseudo-chromosomes.** Scores plotted for ‘Cadenza specific homoeologous homozygote SNPs’ found in the bulk segregant dataset. Scores calculated per 500,000bp window along each chromosome at 10,000bp intervals.
