# Supplementary material for: Mapping‐by‐sequencing in complex polyploid genomes using genic sequence capture: a case study to map yellow rust resistance in hexaploid wheat
Source: Plant J. 2016 Jul 18;87(4):403–19. doi: 10.1111/tpj.13204 (PMC5026171; doi:10.1111/tpj.13204)
Supplement: Supplementary file 2 — Table S1. Summary of mapping statistics across the pseudo‐chromosome reference Table S2. Detailing gene regions within the peak interval 13 650 001–14 150 001 bp on the Genome Zipper‐based pseudo‐chromosome 7 Table S3. Detailing homozygous SNP alleles, in 23–43% of reads, in the bulk segregant dataset within the peak interval (13 650 001 and 14 150 001 bp) Table S4. Detailing gene regions within the peak interval 7 650 001–8 150 001 bp on the MIPS‐based pseudo‐chromosome 7 Table S5. Detailing homozygous SNP alleles, in 23–43% of reads, in the bulk segregant dataset within the peak interval (7 650 001 and 8 150 001 bp) [file TPJ-87-403-s002.docx]

**SUPPLEMENTARY DATA**

**Table S1. Summary of mapping statistics across the pseudo-chromosome reference.** Mapping depth of coverage of the pseudo-chromosome reference and SNP numbers that were identified for the purebred parental lines Avalon and Cadenza plus the bulk segregant dataset. SNPs scored at a minimum quality of 15 and a depth of 30 (for mapping using the POPSEQ chromosomal pseudomolecules directly SNPs were called at a minimum depth of 5). A minimum alternate allele frequency of 10% was used for all SNP calls.

| **Sample** | **Percentage of reference mapped** | **Average depth of coverage** | **Median depth of coverage** | **Number of SNP**  **calls** |
| --- | --- | --- | --- | --- |
| **MIPS Genome Zipper based pseudo-chromosomes** | | | |  |
| Avalon | 97.1 | 40.0 | 32.9 | 2,020,973 |
| Cadenza | 97.3 | 44.7 | 37.3 | 2,280,010 |
| Bulk segregant dataset | 98.2 | 57.5 | 50.3 | 2,780,495 |
| **POPSEQ based pseudo-chromosomes** | | | | |
| Avalon | 97.7 | 42.3 | 34.1 | 1,921,086 |
| Cadenza | 97.8 | 47.1 | 39.0 | 2,142,680 |
| Bulk segregant dataset | 98.6 | 60.2 | 52.6 | 2,571,325 |
| **POPSEQ chromosomal pseudomolecules** | | **e** |  |  |
| Avalon | 28.9 | 4.9 | 2.0 | 2,555,673 |
| Cadenza | 27.1 | 5.6 | 2.0 | 2,760,467 |
| Bulk segregant dataset | 40.9 | 6.4 | 2.0 | 4,604,911 |

**Table S2. Detailing gene regions within the peak interval 13,650,001-14,150,001bp on the Genome zipper--based pseudo-chromosome 7.** Taken from BLASTN alignments over 1000bp long with an e-value less than 1e-5 and identity greater than 80%.

| **Chromosome** | **Start Position** | **End position** | **Associated gene** |
| --- | --- | --- | --- |
| 7 | 13,661,575 | 13,666,347 | *B. distachyon* GDT1-like protein 1, chloroplastic (LOC100846494), mRNA |
| 7 | 13,671,598 | 13,672,587 | *T. aestivum* insertion sequence GeST DNA, complete sequence |
| 7 | 13,674,779 | 13,700,582 | *B. distachyon* disease resistance protein RPM1-like, transcript variant X2, mRNA |
| 7 | 13,703,847 | 13,704,980 | *B. distachyon* premnaspirodiene oxygenase-like, mRNA |
| 7 | 13,725,771 | 13,739,999 | *B. distachyon* disease resistance protein RPM1-like, mRNA |
| 7 | 13,745,548 | 13,749,400 | *T. aestivum* RGA3 (rga3) mRNA, partial cds |
| 7 | 13,756,980 | 13,759,981 | *T. aestivum* strain CN 111380 wheat leaf rust resistance Lr21 pseudogene, partial sequence |
| 7 | 13,773,200 | 13,778,547 | *B. distachyon* putative disease resistance protein RGA4 (LOC100832361), mRNA |
| 7 | 13,779,187 | 13,781,608 | *T. aestivum* Wknox1d gene for KN1 homeobox protein, complete cds |
| 7 | 13,794,300 | 13,796,753 | *T. aestivum* (subclone pAWJL172) AWJL172 gene |
| 7 | 13,797,324 | 13,798,452 | *T. aestivum* clone BAC 1354M21 cytosolic acetyl-CoA carboxylase (Acc-2) and putative amino acid permeases genes, complete cds |
| 7 | 13,812,193 | 13,819,982 | *T. aestivum* glyceraldehyde-3-phosphate dehydrogenase (GAPC12) mRNA, partial cds |
| 7 | 13,827,779 | 13,833,647 | *T. aestivum* cultivar Chinese Spring hexose carrier, LR34, cytochrome P450, lectin receptor kinases, and cytochrome P450 genes, complete cd |
| 7 | 13,852,024 | 13,853,689 | *T. aestivum* cultivar BR34 clone 020713A5 actin gene, complete cds |
| 7 | 13,961,857 | 13,965,894 | *T. aestivum* cultivar K68 triticin gene, complete cds |
| 7 | 13,967,389 | 13,969,473 | *B. distachyon* GDSL esterase/lipase At5g03610-like (LOC100837909), mRNA |
| 7 | 13,969,620 | 13,980,021 | *B. distachyon* ankyrin repeat-containing protein At5g02620-like (LOC100838210), mRNA |
| 7 | 13,981,147 | 13,982,857 | *B. distachyon* clavaminate synthase-like protein At3g21360 (LOC100838820), mRNA |
| 7 | 13,990,651 | 13,998,751 | *B. distachyon* protein ENHANCED DISEASE RESISTANCE 2-like, mRNA |
| 7 | 14,000,374 | 14,003,318 | *B. distachyon* LRR repeats and ubiquitin-like domain-containing protein At2g30105-like |
| 7 | 14,049,353 | 14,052,337 | *T. aestivum* senescence-associated protein (SAP) mRNA |
| 7 | 14,052,745 | 14,059,662 | *B. distachyon* GDSL esterase/lipase At1g71250-like (LOC100824244), mRNA |
| 7 | 14,070,749 | 14,076,528 | *B. distachyon* putative receptor protein kinase ZmPK1 (LOC100825813), transcript variant X2, mRNA |
| 7 | 14,094,391 | 14,098,160 | *T. aestivum* sucrose-phosphate synthase 9 mRNA |
|  |  |  |  |

**Table S3. Detailing homozygous SNP alleles, in 23-43% of reads, in the bulk segregant dataset within the peak interval (13,650,001 and 14,150,001bp).** These SNPs are conserved with unique homozygous SNP alleles, in 23-43% of reads, which were found in the Cadenza parent.

| **Chrom** | **Position** | **Alternate**  **Allele** | **% Reads containing alternate allele** | | **Sequence upstream and downstream of SNP (+/- 50bp)** |
| --- | --- | --- | --- | --- | --- |
| 7 | 13650470 | G | | 35.21 | ACTTAAATGCACCCTGAAAAAGTAATACATGGAGCAATCAAAGTTTCTT[G/A]TGCCATGTTGCTATCTTTAGTCCTCACGTATTTTATTCACCAATGGGAATG |
| 7 | 13653491 | A | | 23.27 | ACGATACCATGTCTTGTGCCTTGGATTTGAACTTACACGAGGAGGCTCT[A/C]GGGATGAACTGGGCAAATTCGCCTGTTTAGGAGCTACCTTGGGAGGCATAG |
| 7 | 13655637 | A | | 26.92 | TGCAGAACATACTCTTACAATGAAAGAAGTGTCAAATGCAAGATAGGCA[A/G]TAGAATCAAGAAGAAATGGCTTACCTTGTGAGTGAGTTTACAGCTAGTATC |
| 7 | 13660401 | G | | 33.80 | GTTTTGTCTCTCTCAGAATATTATCCTCCAACTGAAAACAACATGTTTT[G/A]CGAATACTCGCTTCGCGATCTCTGATCATAAACAACTTTGACCATTTTGTG |
| 7 | 13660416 | A | | 41.18 | GAATATTATCCTCCAACTGAAAACAACATGTTTTACGAATACTCGCTTC[A/G]CGATCTCTGATCATAAACAACTTTGACCATTTTGTGCGTCTACGTGAATAT |
| 7 | 13672430 | G | | 29.07 | AAGCATGCTCCCAGGCTGGATCTTTTCGTGTTCTTCCGGTTGCGGCAGC[G/A]GAAGAAGCCCCTGATCCTACTGATGACATTGTGCTAGTAGCCTACTACTGC |
| 7 | 13677450 | G | | 40.28 | CAGGTCAGAGGCATCAGAGAGTGCGAAGCTATTGTTCCAGTGACTGTTC[G/T]TGTACGTCATATGCTTTCAATGGAACATGCTTACTGTGGTCTGTGGAGCTG |
| 7 | 13677456 | A | | 33.33 | AGAGGCATCAGAGAGTGCGAAGCTATTGTTCCAGTGACTGTTCTTGTAC[A/G]TCATATGCTTTCAATGGAACATGCTTACTGTGGTCTGTGGAGCTGCACAAA |
| 7 | 13677457 | G | | 33.85 | GAGGCATCAGAGAGTGCGAAGCTATTGTTCCAGTGACTGTTCTTGTACG[G/T]CATATGCTTTCAATGGAACATGCTTACTGTGGTCTGTGGAGCTGCACAAAC |
| 7 | 13677461 | T | | 36.51 | CATCAGAGAGTGCGAAGCTATTGTTCCAGTGACTGTTCTTGTACGTCAT[T/A]TGCTTTCAATGGAACATGCTTACTGTGGTCTGTGGAGCTGCACAAACTCAC |
| 7 | 13677473 | T | | 38.46 | CGAAGCTATTGTTCCAGTGACTGTTCTTGTACGTCATATGCTTTCAATG[T/G]AACATGCTTACTGTGGTCTGTGGAGCTGCACAAACTCACTGTAGGGATCAT |
| 7 | 13678233 | G | | 30 | CTATCAAAAGGCTAAAAGACCTTGGTCAAGGAGAGAAGCAATTCCGAGC[G/C]GAAGTGCAAACAATTGGAATGATCCAACACATCAATCTTGTCCGTTTATTG |
| 7 | 13680058 | C | | 32.88 | CAAGGAAGGGGAGTTTACCTACTTCCCAATCTTTGCTGCGGTCAAGGTG[C/A]ATGAAGGGGATGTTGTGTGCCTGCTGGACAGTAGGCTGGCGGGCGATGCGG |
| 7 | 13680066 | A | | 25.68 | GGGAGTTTACCTACTTCCCAATCTTTGCTGCGGTCAAGGTGAATGAAGG[A/G]GATGTTGTGTGCCTGCTGGACAGTAGGCTGGCGGGCGATGCGGATTTGGAG |
| 7 | 13680112 | G | | 26.83 | AGGGGATGTTGTGTGCCTGCTGGACAGTAGGCTGGCGGGCGATGCGGAT[G/T]TGGAGCAGCTGACCAGAGCTTGCAGAACTGCATGCTGGTGCATTCAAGATG |
| 7 | 13680129 | G | | 25.47 | TGCTGGACAGTAGGCTGGCGGGCGATGCGGATTTGGAGCAGCTGACCAG[G/A]GCTTGCAGAACTGCATGCTGGTGCATTCAAGATGATGAGGATCACAGGCCA |
| 7 | 13684538 | C | | 32.89 | TCATAGCATCTATCTGTGAATTGCCTTGCGCCTTCTGTGCGCCATGCGC[C/T]GTGTTGAGTGCGTTTTGGGCTTGTTGTGTTGTGCCCACAGCTCGGACCCTC |
| 7 | 13686827 | G | | 35.19 | TTCCTGAGCTTGGTAAGATGGACCGCCAATAGTGGCAACTTGTTCTGAA[G/A]CCCATGCAGTTTAAGACTCCGTAGGTTCTTCCAGAGCAGGGAGATCCCATC |
| 7 | 13686846 | G | | 30.30 | GGACCGCCAATAGTGGCAACTTGTTCTGAAACCCATGCAGTTTAAGACT[G/C]CGTAGGTTCTTCCAGAGCAGGGAGATCCCATCCAAGCAACTTTGACTGTCC |
| 7 | 13686849 | C | | 30.88 | CCGCCAATAGTGGCAACTTGTTCTGAAACCCATGCAGTTTAAGACTCCG[C/T]AGGTTCTTCCAGAGCAGGGAGATCCCATCCAAGCAACTTTGACTGTCCTTG |
| 7 | 13686852 | C | | 28.17 | CCAATAGTGGCAACTTGTTCTGAAACCCATGCAGTTTAAGACTCCGTAG[C/G]TTCTTCCAGAGCAGGGAGATCCCATCCAAGCAACTTTGACTGTCCTTGTCG |
| 7 | 13686867 | C | | 39.73 | TGTTCTGAAACCCATGCAGTTTAAGACTCCGTAGGTTCTTCCAGAGCAG[C/G]GAGATCCCATCCAAGCAACTTTGACTGTCCTTGTCGAGTCGCACTAACAAA |
| 7 | 13687809 | A | | 25.93 | ACTGCTCTGCTTTCTCTTCTGCCGATATTTCATCGCTATCCTTGGAGTA[A/C]CCCTCTGCAATCCACCTCCTTACCAGTCGCCTACGCCGAATGTTATAGTCC |
| 7 | 13687869 | G | | 25 | TCCACCTCCTTACCAGTCGCCTACGCCGAATGTTATAGTCCCGAGGAAA[G/A]ATTGATAGGTAAAAGATACATGGCTTGAGTGAATCTGGGCAATTACGGAAG |
| 7 | 13688052 | C | | 24 | AAGAATGTATATTATCCATCAATTTCACCGTCTGTGTGGCCAATAATTC[C/A]GCAAGAGCAACTATCACTTTGGGAAGACCTCCACACTTGAAAATAAGTTCA |
| 7 | 13731758 | G | | 36.54 | TCCCGACCACGGAGCCTGCACCAGCTGCCGGACCAGCAGCCACGGCAAC[G/C]GACAAGGTCATGTCGAGCGCGGCGAACCTTGCGCAGCTCCTGCCGACGGGC |
| 7 | 13739416 | T | | 32.69 | GTGACAAGGAAAAGCAGCAGAAGAAGGAGCTCGTCCCATGTTTTGACGA[T/G]GCCATCCGTCTCCTCCCTGTTAAGGATCTTCAATCGGCGGCCACCAAAGTA |
| 7 | 13739467 | G | | 38.46 | CCATCCGTCTCCTCCCTGTTAAGGATCTTCAATCGGCGGCCACCAAAGT[G/A]CGTGATTGCCTCCTTCAAAATTTGTCGACCGAGGAGAGTGCGGCCGTCTGC |
| 7 | 13739566 | A | | 32.35 | GCATGGAGATGCTCCTCCGTGCTCTTCGGGATCATAGCCATGCCCGCCC[A/G]GCGGTCCAGAAGACCAAGAAAGAACTGATGAAATTAGTGGATGAAGCGGAC |
| 7 | 13739662 | T | | 39.34 | CGGACACGGACATCCAAGATTTTCCCACTCAAGTCATGATCTTCTGCTA[T/C]AGCAAGCTGTCCAGGAGCTACAAGAGCTGCTTGCAGTATCTCTATGCCTTC |
| 7 | 13740189 | T | | 32.58 | TGATAAACTTCCTCAAGTCTCTCGACCAAACCTACCGGCTAAATGTGCT[T/G]GATCTTGGGGGTTGCAAGGGTCTGAAGAAGAGCCACCTCAAGAGCATCTGC |
| 7 | 13740288 | A | | 23.94 | GCAAGGTGGTCTCGCTCAAGTACCTCAGCCTCAGGAACACGGACGTCTC[A/C]CACCTGCCCGAGGAGATAAACAATCTCATACTGCTGGAGACGCTCGACATC |
| 7 | 13740355 | C | | 30.51 | AAACAATCTCATACTGCTGGAGACGCTCGACATCCGGCAAACTAAGGTA[C/T]GGGGCCCAGACATGAAGCACATTACCTCCGAAAGCTAAAGCACCTGCTCAC |
| 7 | 13740408 | A | | 33.33 | GCCCAGACATGAAGCACATTACCTCCGAAAGCTAAAGCACCTGCTCACC[A/G]GCCCGAAGATGACCACGGAGGAGGAGACGATCCGCGGGGCAGGGATGCCTC |
| 7 | 13740426 | A | | 40.98 | TTACCTCCGAAAGCTAAAGCACCTGCTCACCGGCCCGAAGATGACCACG[A/G]AGGAGGAGACGATCCGCGGGGCAGGGATGCCTCGCTTGATCGGAAAGATGG |
| 7 | 13740479 | G | | 42.19 | AGGAGACGATCCGCGGGGCAGGGATGCCTCGCTTGATCGGAAAGATGGA[G/A]GACATGGAGATACTGTCCCGGGTCCAGGTTCAACACGGCATGAAGGAACTC |
| 7 | 13740560 | A | | 37.97 | AACACGGCATGAAGGAACTCGAAGAAGTTGCCCGCCTGCTGAAGCTTAG[A/G]AAGCTGGGCGTGGTTCTCATTGGCAGCCAAAGCCAAGCTCAAGACAACATG |
| 7 | 13742210 | T | | 32.95 | AAATTAGAATAGAAGATGCTTGCAGCTATGAAGAGGAGGAACAAGAAAA[T/C]AGAGGAAAGCTTATTAAGAGCAGAGTAGTAATGTGCCAAAGGAAGGTTGGT |
| 7 | 13742799 | T | | 29.87 | ACGATCTGAAAACAAGACAACAATTGTTTGTGTAATGCAAGGGCGGATG[T/C]GACTCTGTAATGTCTTTCTGGCATAAAAAGGCACCACACAAACACAAACAC |
| 7 | 13742880 | A | | 33.33 | CACCACACAAACACAAACACACACAGTTTCTGTCATTCAAGCTCCACAA[A/C]TTAATATTGGCACAAACATCGTATAAACCAACCGGAGGTAGGGATTATGTT |
| 7 | 13742912 | T | | 33.33 | TCATTCAAGCTCCACAACTTAATATTGGCACAAACATCGTATAAACCAA[T/C]CGGAGGTAGGGATTATGTTAAATGAATAACGATTGAATAAACACACACGAA |
| 7 | 13743096 | T | | 30.11 | CGTAACAAGCATGCACACATACGTTTACATGCACACATACTTATTCAGG[T/C]ACGACACACAAATAGATCGAGAGAGAATCGGTCACGCAACACATAGCTCAT |
| 7 | 13743171 | A | | 23.49 | AATCGGTCACGCAACACATAGCTCATGGCTCATGCCTCGGTTTGCACCC[A/G]TTGCTGTGTGGAATGAAGCTCGAGTCGGTGAAGTGGCCCTTGAGCTCCACC |
| 7 | 13743516 | A | | 40.12 | TGAGCTTGTGCTCGTTGTACGAGTTGCGACGGAGCCGAAGGAAGTGCAA[A/G]TGATGGAGATTGTTGCCAAGGTCCTGCAGAGAGTCTTAGATAGAAAAGTGT |
| 7 | 13744751 | G | | 29.09 | CCAACTCAAATTTCTGAGAAACATGAACCCACATGACAATCTCGAAATG[G/A]CCATCCATATTCTCTTGCTTCTGCTTTTTCTCCTGATCATAAACACAACGT |
| 7 | 13747104 | G | | 38.10 | TTACCGACATCCTTGTTGCACCAGACATCATCTAGTACTAGAAGGAATC[G/T]TTTTCCATCTAGTTTCCTCTCCAATTCCTTTTCCAAGGTATTCAGGTTATG |
| 7 | 13752763 | G | | 25.61 | TGATTTGATTAATGCACCGTCCTGCTCTGCAGAAAGTGCCCAATAATAA[G/A]GTAGCCGTGTAAACATCCTGTCAAAAATAATTAGCCGTTAAGCATGGCACT |
| 7 | 13752783 | A | | 27.91 | CCTGCTCTGCAGAAAGTGCCCAATAATAAAGTAGCCGTGTAAACATCCT[A/G]TCAAAAATAATTAGCCGTTAAGCATGGCACTTAATTTACGCCTGACAGTAA |
| 7 | 13754893 | C | | 24.73 | TAATGGCGGATCCAGTGACCATGCTAGGCATGAAAGCCATAGGATGGGT[C/A]ACAGCGCCTATCGTCTCTGAGCTTTTCAAGAAATGCGCCAACTACCTGAGC |
| 7 | 13776813 | T | | 23.86 | ATTGTTCTCATCAGGTGCATATGCCTTGACATGGTCGATATGACTGAAC[T/C]TATCCCACTCAGGGCCAGATTGTCCCGCGGCTATGGAAGTGAGCAACGTCA |
| 7 | 13776903 | T | | 23.53 | GAGCAACGTCAGGCTGCAGCGTAGCTCAAAATGCCTTGGGTTTACATCT[T/C]GCATGTATTCAGGAAGTGTTTCCATGGCATAATCCTCCAATACGAGCCTCT |
| 7 | 13778333 | G | | 27.5 | TCACATGCAATCTTGGGAATGCAATAACCACCTGGGAAGGATGAGGGCT[G/A]TGCTGATGGTAGGATTGCAGGAAATCTGGTCCAACACGCTTGATGGCTGGA |
| 7 | 13803920 | C | | 26.25 | CGGCCAGCCATAGAACCAATGTAAGTCTCTATAGTATGTTGAAAGCCAA[C/G]TATAATGGGAATAACCGGTGGAGAAAGTTGCAACAGTATGTGGCTTTCCTT |
| 7 | 13803957 | C | | 30.43 | GTTGAAAGCCAAGTATAATGGGAATAACCGGTGGAGAAAGTTGCAACAG[C/T]ATGTGGCTTTCCTTCCCGCTAATGTATCCAAGAATTGTGTAAAACCCCATC |
| 7 | 13803966 | C | | 27.78 | CAAGTATAATGGGAATAACCGGTGGAGAAAGTTGCAACAGTATGTGGCT[C/T]TCCTTCCCGCTAATGTATCCAAGAATTGTGTAAAACCCCATCTTGTGTATT |
| 7 | 13803985 | T | | 23.38 | CGGTGGAGAAAGTTGCAACAGTATGTGGCTTTCCTTCCCGCTAATGTAT[T/C]CAAGAATTGTGTAAAACCCCATCTTGTGTATTATCACCAGTAACAGCAAAA |
| 7 | 13841744 | C | | 24.27 | ATGGCCCGTCTCATTCTCATTGCAGGAACACAAACCTCGGGTAGCACAA[C/T]CTTGTTGATCAAGCTCTTGTGGTTGGCGTCCCCCAGAGTCGATCGCGCCAC |
| 7 | 13841753 | C | | 26.53 | CTCATTCTCATTGCAGGAACACAAACCTCGGGTAGCACAATCTTGTTGA[C/T]CAAGCTCTTGTGGTTGGCGTCCCCCAGAGTCGATCGCGCCACCCCACGCCT |
| 7 | 13841756 | G | | 27.96 | ATTCTCATTGCAGGAACACAAACCTCGGGTAGCACAATCTTGTTGATCA[G/A]GCTCTTGTGGTTGGCGTCCCCCAGAGTCGATCGCGCCACCCCACGCCTGGT |
| 7 | 13886398 | T | | 24.69 | GGATCAATGTACCAAGAATCGAATGAAGATGACTTAGATGCATTTGATG[T/C]GTACCCAACTACTGAAGCGGGTCCAATAGATGATGAATCCGAGTCTGACGG |
| 7 | 13898673 | A | | 29.31 | CTGCACACCCCTGATCTCATACACATGTACAACTGGGGTAAGTATGTCC[A/G]GGAGGAAGTGCTTCTATCAGCTGGGAGGGTCAAGTCAGAGCTCCTTGGGGG |
| 7 | 13911254 | G | | 24.59 | CACACCCGCCTGTGTCGGCACTACATCACATTTGTAGGGTTATGGGAGG[G/A]GCGGGGGCGAATAAGAAATCAGTCTTCAGTATGGACCGTAAAAATATTGCA |
| 7 | 13913929 | A | | 31.03 | AGAAATTCAAGATCATTGAACACTCTGAATCCCAGATTCTGAGCTTTAT[A/T]CCTTCATCATTATTCTGTGCTTTAGTTGAACAATAATTAATGCCGGTGGCC |
| 7 | 13967762 | G | | 26.32 | TCGGTGAAAGACGTCGCGGCAGTGGAGCTACCCTACGGCTCTTATCGCA[G/A]CTCCAATTGGTCTGGCGCTCCTGTTCCAACAGGACGCTTCTCCAACTACCG |
| 7 | 13968023 | T | | 35.38 | GGATGTTGGGCCTCGCTGAAGCCCCTCCAGCGTACGAGCTCACATCAGA[T/C]CAATCTTGCGACTCATCTGGCATGACCTTCGCTTTTGGCGGCGCTGGTGTG |
| 7 | 13968035 | T | | 30.15 | TCGCTGAAGCCCCTCCAGCGTACGAGCTCACATCAGACCAATCTTGCGA[T/C]TCATCTGGCATGACCTTCGCTTTTGGCGGCGCTGGTGTGTTCAAGGTGACG |
| 7 | 13968036 | G | | 30.15 | CGCTGAAGCCCCTCCAGCGTACGAGCTCACATCAGACCAATCTTGCGAC[G/T]CATCTGGCATGACCTTCGCTTTTGGCGGCGCTGGTGTGTTCAAGGTGACGT |
| 7 | 13968153 | A | | 36.19 | GCCGACCCTTGCTGCACAGGTTCAAGCTTTCAAGAGGCTAGTCAACGAC[A/G]ATGTCATCTCCACACGACAGCTTCACCACTCTGTCGCACTCATCGCCATCT |
| 7 | 13987253 | T | | 35.71 | CAGAGTGATTGGGAGCCTGCGCTATCTTGTGAACACAGCCTCTCCACCA[T/C]CGCCGCCGCCACCGGAACCACCTGCGCTTGTACTCCCACCATCACCACCAT |
| 7 | 13990669 | T | | 33.09 | TATGGAATGCTCTTCTGTGTCTGTCTGTGCAGTGCTGTTTCGGAGGGTG[T/G]CAGGGATGTCGTCGTCTTCGTCGACCGTGGTCTACGAGGGGTGGATGGTCC |
| 7 | 13990704 | T | | 40.54 | TGTTTCGGAGGGTGGCAGGGATGTCGTCGTCTTCGTCGACCGTGGTCTA[T/C]GAGGGGTGGATGGTCCGGCATGGCCGCCGCAAGATCGGCCGCTCCTTCATC |
| 7 | 13991111 | C | | 30.26 | TACCAAAGCCATCCATTCTATTCCTTGTTCCTCTCATCATACACTTAAC[C/T]GTAATAATCAGCTTGTTTTGTTATTGCACATCCATTTAAGCTATCAACCTT |
| 7 | 13991112 | A | | 26.67 | ACCAAAGCCATCCATTCTATTCCTTGTTCCTCTCATCATACACTTAACT[A/G]TAATAATCAGCTTGTTTTGTTATTGCACATCCATTTAAGCTATCAACCTTC |
| 7 | 13991240 | A | | 39.22 | ATTAGACTATTACTACTTTATTTGCCACAGCTGTATGATTTATTCATTT[A/G]GAGGAAGTTCTTCTATTAGTAAGAAAAAGAACATGTCCTGCAAGTCTGCTG |
| 7 | 13992689 | G | | 35.29 | AAAAAAATCTGAACTCTTCAAGTTTGTGTACCTGATACACCTTATGCAT[G/A]TGTGGTGATGCTTCTGAGTAACTTTGTTTACATTTGTTCGATTGTAACATG |
| 7 | 13992691 | C | | 34.62 | AAAAATCTGAACTCTTCAAGTTTGTGTACCTGATACACCTTATGCATAT[C/G]TGGTGATGCTTCTGAGTAACTTTGTTTACATTTGTTCGATTGTAACATGGT |
| 7 | 13992732 | C | | 38.98 | ATGCATATGTGGTGATGCTTCTGAGTAACTTTGTTTACATTTGTTCGAT[C/T]GTAACATGGTAGAATATTGTTTGTTGTAAAATGACAGTTCCCTTAATTCTT |
| 7 | 13992873 | C | | 38.46 | TAATCACCGATCATTGATGCGAAGAACAACAATTGGGAATGGTCAGTGA[C/T]ATGGTCTGTTTGTTGATTGTTCCTCCATGTATGTTTTTTGTTCCAGTGCTT |
| 7 | 13992902 | A | | 40 | CAATTGGGAATGGTCAGTGATATGGTCTGTTTGTTGATTGTTCCTCCAT[A/G]TATGTTTTTTGTTCCAGTGCTTAAAGGATTTCTATATTCTTGGCATTTGTA |
| 7 | 13992933 | G | | 41.90 | TGTTGATTGTTCCTCCATGTATGTTTTTTGTTCCAGTGCTTAAAGGATT[G/T]CTATATTCTTGGCATTTGTAGGTCCTCCGGAATCATTGCATGATTGGACTC |
| 7 | 13993115 | C | | 38.10 | AGAATGGCAAGAACTTTCTTTGTCCCTTGCTTTGGTTTTCTGTTGTAAC[C/T]GGGCTGCTTGTTCAATGGGTGTTAAATTTGAACTTTACTTCAGGTCTCCGC |
| 7 | 13993116 | A | | 37.65 | GAATGGCAAGAACTTTCTTTGTCCCTTGCTTTGGTTTTCTGTTGTAACT[A/G]GGCTGCTTGTTCAATGGGTGTTAAATTTGAACTTTACTTCAGGTCTCCGCA |
| 7 | 13993129 | G | | 28.21 | TTTCTTTGTCCCTTGCTTTGGTTTTCTGTTGTAACTGGGCTGCTTGTTC[G/A]ATGGGTGTTAAATTTGAACTTTACTTCAGGTCTCCGCATTTTTGAGGAGCT |
| 7 | 13994091 | A | | 32 | CAACAACATAGCCTCCTTCATATGCTGAACAGCGTTGCCGGTACAATTT[A/T]TTGTCTTTGGAGTAAACTTTTGAACAACATTTCTAAGATATTCCTATTGTT |
| 7 | 13994429 | C | | 26.37 | TGTCCTGGGTGAATGAAGTGGTGATGTTTGCGATAAATCATGTTAAGTC[C/T]ATTTCTACCTTAGGTCTTACCAGGTCATCACTGTAGGATTCCTACAGTTTT |
| 7 | 13994435 | C | | 26.67 | GGGTGAATGAAGTGGTGATGTTTGCGATAAATCATGTTAAGTCTATTTC[C/T]ACCTTAGGTCTTACCAGGTCATCACTGTAGGATTCCTACAGTTTTCACAGC |
| 7 | 13995096 | T | | 24 | GCACAAAAAACGCAGGATACAACCGTATGGTTATTTACTTTCTTGACTG[T/C]GGACTTATCAATAATATCAAGCCTCAAATATGAACTCACTTGAACAGCGCA |
| 7 | 13995120 | C | | 33.33 | GTATGGTTATTTACTTTCTTGACTGCGGACTTATCAATAATATCAAGCC[C/T]CAAATATGAACTCACTTGAACAGCGCATCCTTTCTTTTAGCAACATGATCC |
| 7 | 13995144 | T | | 27.27 | GCGGACTTATCAATAATATCAAGCCTCAAATATGAACTCACTTGAACAG[T/C]GCATCCTTTCTTTTAGCAACATGATCCATTCGTTTCACATCTTTAAACCAG |
| 7 | 13998180 | A | | 41.86 | AGGAGATGCTTGCCGACGTTGCAACCACCACTTGGACCAAATGGACGAT[A/G]GTAGTAATCTGGTTAATCCTCAAGCACAGGGACAACCGCAGATGATAGTTC |
| 7 | 13998354 | C | | 33.33 | TCAGTTTAGCCAATGGTTCGGTCCTGCATCAGCGACGAGCTTGAGAGAG[C/T]ATTTCTTAGATGGCAAATGTATGTGTACCTGGACAAGAAAAGCCATGTCGA |
| 7 | 14000312 | A | | 25 | TTGCACATTTCTATTCTCTATTTGCTCGCCTCTTCTTTTTAATCTTGGC[A/G]ACATTTATACTGAAGAGAACATACCGTACTATTCGATGTTTGTAAGAAGTT |
| 7 | 14000698 | A | | 23.08 | AGCTCTGTTTGTTTCTGCAATCTCTCTTACTGCTGCTTTCTATAGCTGG[A/G]TTCGCCCTGGGACACTTCCACATAATATAGTTTCCATGTTTATACCACTTG |
| 7 | 14037633 | C | | 23.33 | TTGCATTGCCAAATGCGCCTATCTCTGCAACACAAGCATCAAAAGAGAA[C/T]CCAGATGTGTCTAAAACCCTTCTTCTTTATACTAGATGGATCCACAATACA |
| 7 | 14060552 | A | | 40.96 | GTCTCCAAATTCCACAGAGAAGCCGGTGGAAGAAGAATCAACAATGGCC[A/G]CAAGGTATCTTTGGCCTTGGACCCGCCGGCTGGCCGGCCGGGATATACATA |
| 7 | 14060554 | G | | 41.46 | CTCCAAATTCCACAGAGAAGCCGGTGGAAGAAGAATCAACAATGGCCGC[G/A]AGGTATCTTTGGCCTTGGACCCGCCGGCTGGCCGGCCGGGATATACATACA |
| 7 | 14060555 | T | | 40.96 | TCCAAATTCCACAGAGAAGCCGGTGGAAGAAGAATCAACAATGGCCGCA[T/A]GGTATCTTTGGCCTTGGACCCGCCGGCTGGCCGGCCGGGATATACATACAT |
| 7 | 14061706 | A | | 38.14 | TGTACACGTCTCTGTACAGTGTACATGCAGCATCGACCGATATTCCGGC[A/G]GCCGGCCGGTGGAGATGTGCGTGTCTGGCGTCGCCGTTGGCGTACGTGCGG |
| 7 | 14061756 | A | | 25.97 | GCCGGCCGGTGGAGATGTGCGTGTCTGGCGTCGCCGTTGGCGTACGTGC[A/G]GGCCGGCTCCGATGGACCATCGATCGGTTACTGGCTCATCCGTGGATGCAT |
| 7 | 14064326 | G | | 33.71 | TTTCGGTTTTCGTCCTTCCACTCTACACATTTTCTTGATTTACTTGCAT[G/A]CATGGTTAATGATATCAATGTTTTCAGGGTATGCGTCATCCTTGGGATGGC |
| 7 | 14064389 | T | | 32.43 | ATCAATGTTTTCAGGGTATGCGTCATCCTTGGGATGGCCATGATGATCA[T/C]GCGCATGGTCATGGACATGCACACGAGCATGAGGTATTAGCTTCAGATCAC |
| 7 | 14064419 | G | | 25.33 | GGGATGGCCATGATGATCACGCGCATGGTCATGGACATGCACACGAGCA[G/T]GAGGTATTAGCTTCAGATCACATCTCCACTCTTTTTGTATGTGCTTTAATT |
| 7 | 14064834 | C | | 39.29 | TTAAACCTTGCTGCATAATCATTTCAATGGCATGCTATGTTTATGCGTG[C/T]CTGTAGGGAGTTAGAGCTACCAAGTAGTGGTAATCGATGTTATACATCCTC |
| 7 | 14071502 | C | | 24.07 | CGAATACAAAATTCTATATTGGTATACAAGCGGCTCATGCTATTCTTGA[C/T]TTACATAGATACACATCATAGTTTTGCATCCAACAATAAGTATACATGCTG |
| 7 | 14072112 | A | | 41.07 | TCCGCGATACATTTTTATTTGACCCGCCTCTGTTTAGAAGTTTGGCAAG[A/G]CCAAAGTCGGTGATCTTTGGCTCTAAGTTCTCATCCAACAGTCTGTTCTCT |
| 7 | 14073028 | G | | 25.49 | CATTGCATGTGCAGTCACTCAAGCATATCCGCTTGCAAGCAGGAAGAGA[G/A]ACTGACTCAATCTTACGATCATAGCCTAGGAAGTCCGTGGAAGGTAGCTTC |
| 7 | 14073336 | A | | 24.62 | CCATTTGGCTTATTTAGACTGTATAATCTAAGGTTGCCATCATAATCCA[A/G]TGTTAGCCTTCGCATAACCCTGAGACCCCAATCAGCAGCCTTTATAGTTAA |
| 7 | 14073602 | T | | 24.66 | ATGGAAGCTGTAGTGCCCAGGAACAAGTAGCCTATTACTAGATTCCAAC[T/C]TTGAACCAGCAGTTATATTCTGATTGGGTAGCAATGTATCAGTAGGAGAAT |
| 7 | 14073606 | T | | 24 | AAGCTGTAGTGCCCAGGAACAAGTAGCCTATTACTAGATTCCAACCTTG[T/A]ACCAGCAGTTATATTCTGATTGGGTAGCAATGTATCAGTAGGAGAATCAAA |
| 7 | 14073611 | A | | 25.97 | GTAGTGCCCAGGAACAAGTAGCCTATTACTAGATTCCAACCTTGAACCA[A/G]CAGTTATATTCTGATTGGGTAGCAATGTATCAGTAGGAGAATCAAAGCTTT |
| 7 | 14073619 | G | | 25.64 | CAGGAACAAGTAGCCTATTACTAGATTCCAACCTTGAACCAGCAGTTAT[G/A]TTCTGATTGGGTAGCAATGTATCAGTAGGAGAATCAAAGCTTTGCCATAGA |
| 7 | 14073628 | T | | 27.16 | GTAGCCTATTACTAGATTCCAACCTTGAACCAGCAGTTATATTCTGATT[T/G]GGTAGCAATGTATCAGTAGGAGAATCAAAGCTTTGCCATAGAACTATATCA |
| 7 | 14074069 | T | | 23.53 | TCACCACACAATTGGAAGTTTGAAACAAGTAGGGTATGTTTTTTGACTT[T/A]TCTACTATGTAATGGATGGTCAGATTAGGCAATGAGTGGCCCAAATTGAAT |
| 7 | 14074091 | G | | 24 | AAACAAGTAGGGTATGTTTTTTGACTTATCTACTATGTAATGGATGGTC[G/A]GATTAGGCAATGAGTGGCCCAAATTGAATGATGGCTTGGGAGCCTGGAGCC |
| 7 | 14074477 | A | | 23.53 | TCTCTGCTTCTTCTAAATCTGATTCATCTGTTTTTGCGGATCTCGGCTC[A/G]TGAATTCCTCTCGCCAGGCTCCTCTCTCTCCGTAGAGGATAGCTCTGACGT |
| 7 | 14074579 | T | | 26.15 | CTCCATTCACCAGATGGTACTTTCACCTGCGGCTTCAACAATATTCTGG[T/G]AATGCCTTCGTTTCTCTATTTGGTTCTCCAACACAGCTCATGAAAAGACTG |
| 7 | 14076020 | G | | 35.37 | TGCGGGGCATCTGGTATTGTATACAAGGGGGTCTTGAAAGACAAGAGGT[G/A]GTAGCGGTGAAAAGGTTGGCAGACATAACCAAGGCGAAGAAGAATTCCAGC |
| 7 | 14080130 | G | | 26.47 | GGTTTGCTCCCTATTTATTCTGTAAGACTTGTTCTCATGCTAGTTTCTT[G/A]TTTGTATAGCCTCAGCAGCCTCTAACATTGGTTTTTGTGGAGACAAAACGA |
| 7 | 14083353 | A | | 27.27 | CTAATCTGGAAAGGTAGCGATGCGCTCTCTCTCTCCTCTACAAGTATAA[A/T]CAGGATTAGCATAGCCCAGCAGCCAATCTGGCACTTGTAGATTTGCCTCAA |
| 7 | 14095909 | G | | 38.36 | ATGCTCATTTCCGGTGATCTTTGATGCAGGTGTTGGACAACGGCCTCCT[G/T]GTTGATCCCCACGATCAGCATGCAATCGCAGATGCACTCTATAAGCTTCTT |
| 7 | 14102540 | A | | 31.87 | TGGAGAGGAAGGAAATGATTCAATAGCTCAAATATAAGTTCAATATATT[A/G]TTTGGAACAGGGGACATCCATACCCTCTGTATTGCAATCTTTGCTGTTGGA |
| 7 | 14126783 | T | | 28.17 | ACACCAATCTGACATCAATGAGAAAATGCGCGGCATTCTGGTTGACTGG[T/C]TGATTGAGGTAAGGATTCCATTCGATGCTCGGTGGATTCTGCAAAGTAGTC |
| 7 | 14126806 | T | | 30.26 | AAATGCGCGGCATTCTGGTTGACTGGCTGATTGAGGTAAGGATTCCATT[T/C]GATGCTCGGTGGATTCTGCAAAGTAGTCTTATCTTAGTATGCTCTGGAATG |
| 7 | 14126823 | G | | 30.30 | GTTGACTGGCTGATTGAGGTAAGGATTCCATTCGATGCTCGGTGGATTC[G/T]GCAAAGTAGTCTTATCTTAGTATGCTCTGGAATGGAAAGTTCTTAGAAACC |
| 7 | 14126838 | A | | 30.65 | GAGGTAAGGATTCCATTCGATGCTCGGTGGATTCTGCAAAGTAGTCTTA[A/T]CTTAGTATGCTCTGGAATGGAAAGTTCTTAGAAACCCCAATGTTCTTGTCC |
| 7 | 14131220 | C | | 23.60 | GGACAAGCCCAAACTTAACTACCAAGTAGAACCATTCGCCAACGGGCCA[C/T]GGGTTCAACATGTAATTCATCGGAAAAGGGTGGTTCACATGTCGTTCTTCG |
| 7 | 14132583 | A | | 30.38 | TCAGGCTTTAGCAGTCCTGAAGATCCAGAGCAGCCAGCGCTTCAGTTCG[A/G]AAAGTATTCGATGCTGCAGTGCAGACCGGGCTTCGATCCTGATCCTCCTCA |
| 7 | 14132586 | G | | 30 | GGCTTTAGCAGTCCTGAAGATCCAGAGCAGCCAGCGCTTCAGTTCGGAA[G/A]GTATTCGATGCTGCAGTGCAGACCGGGCTTCGATCCTGATCCTCCTCACTC |
| 7 | 14132588 | A | | 29.27 | CTTTAGCAGTCCTGAAGATCCAGAGCAGCCAGCGCTTCAGTTCGGAAAG[A/T]ATTCGATGCTGCAGTGCAGACCGGGCTTCGATCCTGATCCTCCTCACTCCT |
| 7 | 14135113 | G | | 23.61 | AAGAAATCCTGATAAAATCTTACAGGAAACTGTAAGAACAACAAGCGAA[G/C]TGGAGTTCACATATGAGATGTTGCTAATGAAACAGACCATCAACTAAAGAG |
| 7 | 14138822 | A | | 40.74 | TCAGTGTCTCTGATTTCCTGAAATGTTCAGCTGAGCCTACAGGGGAGCG[A/C]TGTGCAAAACTATGCCGCAACCTGGGTTCTCATCTGAGAGAGAAGGATGAC |
| 7 | 14138825 | C | | 41.51 | GTGTCTCTGATTTCCTGAAATGTTCAGCTGAGCCTACAGGGGAGCGCTG[C/T]GCAAAACTATGCCGCAACCTGGGTTCTCATCTGAGAGAGAAGGATGACTAT |
| 7 | 14138829 | G | | 40.74 | CTCTGATTTCCTGAAATGTTCAGCTGAGCCTACAGGGGAGCGCTGTGCA[G/A]AACTATGCCGCAACCTGGGTTCTCATCTGAGAGAGAAGGATGACTATGACA |
| 7 | 14138831 | G | | 42.31 | CTGATTTCCTGAAATGTTCAGCTGAGCCTACAGGGGAGCGCTGTGCAAA[G/A]CTATGCCGCAACCTGGGTTCTCATCTGAGAGAGAAGGATGACTATGACAAC |
| 7 | 14139169 | C | | 37.5 | CAAGAGACAATACACAAGTGAAGACGTGCAGGACTCATGGCATTATGCA[C/T]GAGTTTGCGCTACACAAGTCCATGTCACAGGGGTTCATCGCGACATCGTCT |
| 7 | 14139368 | A | | 41.38 | CATGTCCGTAAGTGCAAACTGCTGCGAGTCTTGGATCTGGAAGAATGCA[A/G]TGATCTGGAAGACAGTCACCTCAAACACATAGGCAAACTGTGGCATCTGAC |
| 7 | 14139759 | A | | 33.33 | TTCACATGAGAAATCTGAGAAAGGTTAAGATATGGTGTGGCCCAGTTGC[A/C]AATGGAAGTAACTACACCACAGATCTTTCGGAGGCCATTCAGGAATTCACC |
| 7 | 14139779 | A | | 32 | AAGGTTAAGATATGGTGTGGCCCAGTTGCCAATGGAAGTAACTACACCA[A/C]AGATCTTTCGGAGGCCATTCAGGAATTCACCAAAGTCCCCATGGACAGTAT |
| 7 | 14139780 | T | | 37.25 | AGGTTAAGATATGGTGTGGCCCAGTTGCCAATGGAAGTAACTACACCAC[T/A]GATCTTTCGGAGGCCATTCAGGAATTCACCAAAGTCCCCATGGACAGTATG |
| 7 | 14139789 | C | | 36 | TATGGTGTGGCCCAGTTGCCAATGGAAGTAACTACACCACAGATCTTTC[C/G]GAGGCCATTCAGGAATTCACCAAAGTCCCCATGGACAGTATGGACGCCCGT |
| 7 | 14139790 | A | | 36 | ATGGTGTGGCCCAGTTGCCAATGGAAGTAACTACACCACAGATCTTTCG[A/G]AGGCCATTCAGGAATTCACCAAAGTCCCCATGGACAGTATGGACGCCCGTT |
| 7 | 14140367 | G | | 40.79 | AAACAAGACAAGACTGGGAGCAAGCAACAAAGAACCACCCGAATAGGCC[G/A]AGATTCATGTTGCACAAAAGTGATGTTCGAGTGGAAAGCGAGGGACCGGGG |
| 7 | 14140427 | A | | 27.03 | TGCACAAAAGTGATGTTCGAGTGGAAAGCGAGGGACCGGGGAATGAAGA[A/G]GCTTCAGCCGTGAGGGAGAAGAGGAAGATATGTGTAGTCCAACCAAGTTTG |
| 7 | 14140436 | T | | 40 | GTGATGTTCGAGTGGAAAGCGAGGGACCGGGGAATGAAGAGGCTTCAGC[T/C]GTGAGGGAGAAGAGGAAGATATGTGTAGTCCAACCAAGTTTGGATGACGGA |
| 7 | 14140514 | A | | 41.33 | TCCAACCAAGTTTGGATGACGGACTGGATTCTAGTCTCAAGAAGATGAG[A/G]CTTTCATCAGACTCTTCTTCGCGTCTTCAAATGATTGTCCATACGAGTCCC |

**Table S4. Detailing gene regions within the peak interval 7,650,001-8,150,001bp on the MIPS-based pseudo-chromosome 7.** Taken from BLASTN alignments over 1000bp long with an e-value less than 1e-5 and identity greater than 80%.

| **Chromosome** | **Start Position** | **End position** | **Associated gene** |
| --- | --- | --- | --- |
| 7 | 7,663,051 | 7,665,129 | *B. distachyon* E3 ubiquitin-protein ligase KEG (LOC100828572), mRNA |
| 7 | 7,669,366 | 7,671,834 | *B. distachyon* cytochrome P450 71D8-like (LOC100845626), mRNA |
| 7 | 7,680,755 | 7,686,939 | *B. distachyon* protein S-acyltransferase 24 (LOC100832843), mRNA |
| 7 | 7,688,744 | 7,692,711 | *B. distachyon* laminin subunit beta-1 (LOC100829573), mRNA |
| 7 | 7,697,836 | 7,699,224 | *B. distachyon* NF-X1-type zinc finger protein NFXL1 (LOC100843639), mRNA |
| 7 | 7,701,741 | 7,710,797 | *B. distachyon* probable isoleucine--tRNA ligase, cytoplasmic (LOC100833154), mRNA |
| 7 | 7,710,800 | 7,714,152 | *B. distachyon* probable transcriptional regulator SLK3 (LOC100842018), mRNA |
| 7 | 7,722,193 | 7,732,189 | *B. distachyon* U3 small nucleolar RNA-associated protein 21 homolog (LOC100833468), mRNA |
| 7 | 7,738,084 | 7,743,607 | *T. aestivum* mitochondrial acid phosphatase (ACP) mRNA, complete cds; nuclear gene for mitochondrial product |
| 7 | 7,769,011 | 7,773,180 | *T. aestivum* sucrose-phosphate synthase 9 mRNA, complete cds |
| 7 | 7,777,893 | 7,779,872 | *T. aestivum* vacuolar proton-inorganic pyrophosphatase mRNA |
| 7 | 7,860,974 | 7,862,788 | *A. tauschii* chromosome 1Ds prolamin gene locus, complete sequence |
| 7 | 7,853,846 | 7,858,527 | *B. distachyon* dual specificity protein phosphatase PHS1-like |
| 7 | 7,885,679 | 7,887,186 | *T. aestivum* putative disease resistance mRNA, partial sequence |
| 7 | 7,936,847 | 7,938,817 | *Avena strigosa* clone L7M2.3 putative resistance protein gene, partial cds |
| 7 | 7,955,272 | 7,963,235 | *B. distachyon* endoplasmin homolog (LOC100823353), mRNA |
| 7 | 7,976,981 | 7,980,809 | *B. distachyon* E3 ubiquitin ligase SUD1 (LOC100827964), mRNA |
| 7 | 8,022,017 | 8,026,370 | *Oryza sativa* Japonica Group MET1 gene for putative cytosine-5 DNA methyltransferase, complete cds, allele: OsMET1a |
| 7 | 8,040,733 | 8,043,356 | *T. durum* cultivar Italy 363 NBS-LRR protein (RGA-7Ba) gene  *T. aestivum* clone Tp3a5a powdery mildew resistance protein pseudogene, partial sequence |
| 7 | 8,062,633 | 8,066,911 | *Setaria italica* probable protein phosphatase 2C 60 (LOC101765411), mRNA |
| 7 | 8,069,330 | 8,070,665 | *B. distachyon* pentatricopeptide repeat-containing protein At4g15720 (LOC100834971), mRNA |
| 7 | 8,120,921 | 8,123,363 | *B. distachyon* putative receptor protein kinase ZmPK1 (LOC100836294), mRNA |
|  |  |  |  |

**Table S5. Detailing homozygous SNP alleles, in 23-43% of reads, in the bulk segregant dataset within the peak interval (7,650,001 and 8,150,001bp).** These SNPs are conserved with unique homozygous SNP alleles, in 23-43% of reads, which were found in the Cadenza parent.

| **Chrom** | **Position** | **Alternate**  **Allele** | **% Reads containing alternate allele** | | **Sequence upstream and downstream of SNP (+/- 50bp)** |
| --- | --- | --- | --- | --- | --- |
| 7 | 7652983 | T | | 30.91 | TCCCGCCCAGCTCGGTCCTGGCCCGCTTAAAAAAATAGCCGGTCCGGTC[T/C]GTGAAATATGGACCGAAAATTCGTCGGTCCGGTCCGGTTAAAGCCCGGTCC |
| 7 | 7652984 | A | | 37.04 | CCCGCCCAGCTCGGTCCTGGCCCGCTTAAAAAAATAGCCGGTCCGGTCC[A/G]TGAAATATGGACCGAAAATTCGTCGGTCCGGTCCGGTTAAAGCCCGGTCCG |
| 7 | 7653133 | A | | 33.90 | ACGGAGCAATCACATGATAATTTGTTGCTATACCTATATGGGAATAATT[A/G]CCCAGGATTCTTCAATCCATTCTCTCCTTATTTCCACGTACTCCCTCCGTA |
| 7 | 7653146 | T | | 29.82 | ATGATAATTTGTTGCTATACCTATATGGGAATAATTGCCCAGGATTCTT[T/C]AATCCATTCTCTCCTTATTTCCACGTACTCCCTCCGTAAAAGTAGTTACCC |
| 7 | 7663896 | A | | 26.85 | ACCCTCTCCACTGCTGAGGAACGGACCTTCAGAGGTCATGTATTCCATG[A/G]ACTCGTACAAGAATGTAGCTATCGCGTTCTCCATCCTCCATGCCAACTATG |
| 7 | 7664300 | G | | 29.82 | TCAAATGCTTGTAAGATATCTGACATCAAGGGGCGATCCCGAAAGTCGT[G/A]CTCAAAGCAGCTAGAAAGGACATTCTCAACCTCTGCAGGTAAATTGTACGG |
| 7 | 7664929 | T | | 40.26 | GAGAACACCTGCAGCTGGTCGTCCCTGACCGGGTGCAGCATCTTCACAG[T/C]GACCTCGTGGTACCGGTCATAGTCCTCCGTCCTCGGGTGGTGCGTGGCTAT |
| 7 | 7665153 | T | | 25.32 | AGCAGCATGTACTCGAACGATTCGGTGTCGGTGGCTGGGAGCCTGCCAT[T/C]CCTGCAACCACAAAATTCACCATTGGTTTCTCAACAACAGGTGCAGAGTAC |
| 7 | 7667660 | G | | 24.69 | CAACACTCGATCATGCAACCGTGTACCAGGAGACCAGGGCATGAAGGTT[G/C]TTGAATGGTTCCTCTTGTTATCATGCATGTTCAGTTGTGTGGATTTAGAGG |
| 7 | 7667818 | G | | 31.03 | TGGGGTGGGGGGATCACAGGGATGCGTCACAGATGTATTTGTTGTTTGC[G/T]GCCAAATTAAGCGATGATCATGAATGTATAGTGTGTTCCATGCATACGTGT |
| 7 | 7667863 | G | | 36.54 | TTGCTGCCAAATTAAGCGATGATCATGAATGTATAGTGTGTTCCATGCA[G/T]ACGTGTTATGTTTGATTAGTACTTCAGCGGATTTGCTAAGACTAGCCACAA |
| 7 | 7669435 | A | | 25.93 | AGGGACAAGCACGAGGTCGTTGCGCCGGCGCACGGTGACGCCCATCTCC[A/T]CCGCCATGTCCAGCTCCCCGGCCACCGCTCCGGCCGGCAGCTCCCAGTCGA |
| 7 | 7669436 | A | | 26.83 | GGGACAAGCACGAGGTCGTTGCGCCGGCGCACGGTGACGCCCATCTCCT[A/C]CGCCATGTCCAGCTCCCCGGCCACCGCTCCGGCCGGCAGCTCCCAGTCGAA |
| 7 | 7669721 | T | | 26.60 | TCCCTGCTGATGGCCCATGCGTTAACGAGCACCATTGTCCCCTTGGGCA[T/C]GTCAAAGCCTAGGACACGCTCTTGCGCTCCTGTGGCAGCAGCAGCGGCGCC |
| 7 | 7669844 | G | | 31.08 | CCTTGACCACCAGGCGCAGGTAGTTTAGATCGACGAGCAAGGACTCGAT[G/C]ACCATGGGCTGTCCGACGAGCGCGAGTCGGACCTCGTCCTGCGCCTTCGCA |
| 7 | 7669873 | A | | 32.88 | TCGACGAGCAAGGACTCGATCACCATGGGCTGTCCGACGAGCGCGAGTC[A/G]GACCTCGTCCTGCGCCTTCGCATTGTTCTCGGGTTCCTAACAAGCTCAGAC |
| 7 | 7669892 | T | | 28.57 | TCACCATGGGCTGTCCGACGAGCGCGAGTCGGACCTCGTCCTGCGCCTT[T/C]GCATTGTTCTCGGGTTCCTAACAAGCTCAGACATGGCCTACTGCAGCACCG |
| 7 | 7670381 | T | | 25.27 | CGAACAGCTTGGTCTCACGCTGCAACAACGCCAGGAACACGCCCCGATC[T/C]TTGAACTTGCTCCGATGACAGCGCACACTGATGAGTCAGCGGCGTACGCGG |
| 7 | 7670702 | A | | 41.38 | GGTTTGTTGTACAAATAATAGACATGCAATATACATCTAAACACCACAG[A/G]AAGGCCACCACTCGAGCACACATCTCATTTGCCGTCCCACAATAACCCTCT |
| 7 | 7670839 | T | | 42.99 | ATCCACCGTCATTGGCGCCGTGCGGGCTTTGCCAAGCGCCCTGCCTTGG[T/C]GGCAGTGAGAGGGGAATAAAAATGGCTGGTGGGTAGGAATTTTGTTCCCTC |
| 7 | 7670968 | A | | 23.53 | CTTGTACTGTCGTTTATGATTACGTTCATTGTTATGCAATTTTGTTTCT[A/G]TTTTTATAGTGCTGCTATTTTTTCAAATTACAGTACAACTAAAATTCAAAA |
| 7 | 7692554 | T | | 40.79 | ATGAGGGCGTTACCGACAGAATGCTCTGAACATTATCCGAGTCTTCTTA[T/C]GAGCTCATATTTATTGCCTGCACGCAGGAGGGAAGGAACACCGTCTGGCCA |
| 7 | 7696925 | G | | 25.56 | TTTGTCTGCTGGGCTTTTGACATGGAAGTTTTTGAAGAAAACATTTGCA[G/C]GGAAGAAAGTTATTGCTTCATCAACTATATCCGCCTTATCTGTAATAGCAT |
| 7 | 7716319 | C | | 41.30 | CCACCGGTGGATGGTGAATTGCTTGCTGTGCTGACTTGTTTCTTGGCTT[C/G]GTCGAGAAGGTGGCTCGCCTGACGCCGTCGCTGTCGCCGTGGACGACGACG |
| 7 | 7723313 | T | | 39.62 | ACACACAAAATTCAGAAAGCAAACTATCTCCAAAAAGAAATCAATAATT[T/G]CAGGCACCTTATTTAGGTAAGTATCCGGATGCATAATGCAGGTTGGAGTAA |
| 7 | 7723548 | C | | 23.60 | TCTTCATGTTTAGTAAAGGTAACCACCTGATTTTTGAAGAGAAAACATA[C/A]ACATGAAGGCCTCAATACTTCAATAGTAGTTGATGGACATGATATAGTGGG |
| 7 | 7728939 | A | | 28.79 | GTATCTTTGGTAGGCTTTCATTGTTTATTGTTCTCACCCTGTGCATTTG[A/C]ATACCTTTGATAGCTTCATATTTCAGGAAATATCATAAGATGAGTTTCCAA |
| 7 | 7732576 | C | | 30.70 | TCATAAAAAATGCTGCAAGAATAGCCAGTGTCCACACTTGAAGCTGTGT[C/T]ATCTGGTAGTTTGATGAGTTATGTTTTTTGTTATCTGGCTGGAGGGTGTTC |
| 7 | 7734390 | T | | 31.37 | TTATTGAAGAAAGTTGTAAAACTATTTATTGACCAAAACTGCTGTAGAA[T/A]TGCTCAGCATGCTTCTGTCAACTGTATCTATACACGGTTTTCCGATCGATC |
| 7 | 7744666 | T | | 39.44 | ATTACAGAGCTAGTCCATCACCGTTCCAGTTCTTGAATTAATTTCCATG[T/C]ATTGGCTACATTTTGACACTTGGTTTTCTATTTTTATGGGTCGTATCGAGC |
| 7 | 7744675 | A | | 42.67 | CTAGTCCATCACCGTTCCAGTTCTTGAATTAATTTCCATGCATTGGCTA[A/C]ATTTTGACACTTGGTTTTCTATTTTTATGGGTCGTATCGAGCGCAGGTGGA |
| 7 | 7744760 | G | | 28.28 | ATCGAGCGCAGGTGGATCTCAAGTATGAAGACGATCATCCGGTAACCGG[G/A]CAAGGAGTATGCAGAAAGGTGATGAAGCAGCTGCACGAGACCTATGCCTCT |
| 7 | 7745515 | T | | 25.00 | AGGATTGAAACCACTCACACGAAGGCGGAATTTAGGATTGTCGGATTGT[T/C]CGAAAACAGTTGCTATAACCAGACGTAAGATGTTACTCTTGATTTGAATCA |
| 7 | 7745951 | C | | 30.26 | ATTGCAAAGGTATAAAAGGTCTCTGTCCACGTCGCAAAGGTCAAAGCTG[C/G]TGGAGGCATCCAGGCAGAGGCCGGATCAGAGGATGTCGAAGTTGCATGGTG |
| 7 | 7745977 | C | | 31.82 | CCACGTCGCAAAGGTCAAAGCTGGTGGAGGCATCCAGGCAGAGGCCGGA[C/T]CAGAGGATGTCGAAGTTGCATGGTGTAAGTTACTCTGATACCCTGAATTCA |
| 7 | 7746048 | A | | 28.75 | GGTGTAAGTTACTCTGATACCCTGAATTCAGCTAAACTGCGGTAGATAC[A/G]TTCACAACGTCTCCTAAAAACATCAAAAAGACAAAAAATAAATTCTCAATT |
| 7 | 7746374 | A | | 35.71 | GCAGTTAAGCGTTGCAGATGGTAGACAACTATACACACCTAATGGGAGA[A/T]GGAACTTCAATAAAGACGTGAGCAACTGACCATACCTGTTGATTGGACTCC |
| 7 | 7748679 | C | | 36.84 | ACGAAAGAAAGCGAACGACGAACACATGAATCCCGTACGTGGGCCGGCC[C/G]AACCAGATGTTTGCTTCAGCGAAGGGACGACCTTTTTGACGCTAACGAGCG |
| 7 | 7748691 | C | | 30.36 | GAACGACGAACACATGAATCCCGTACGTGGGCCGGCCGAACCAGATGTT[C/T]GCTTCAGCGAAGGGACGACCTTTTTGACGCTAACGAGCGTCAAATAGTATC |
| 7 | 7750088 | G | | 24.14 | TACCACTATCATGGTGTAACATTCATGCAGTGGATCAAAAGCTCATCTC[G/A]TTATCAGTTAATCTGAATATACCTTCAGTGGATATTTCTGTACATAGAGGC |
| 7 | 7756751 | T | | 26.51 | ATTGCTTTTTTTACTCCAACGCATCTGAAGCGGTGATGGGTACTTGTGC[T/G]GAGGCGGCGGAGGCAGCGGCGGCGCTGTATCTAAAGAGGGCACCATAGCAG |
| 7 | 7761049 | A | | 25.37 | ATGTGCGGTGGAGAGAGTCCACCGAGGAGATGAGGTTGCCAGTTCCCAC[A/G]TGCCTTCTCTATCTTGTGGCCATCACGACGGCCAGCGACACAACGACACCA |
| 7 | 7761063 | C | | 30.30 | GAGTCCACCGAGGAGATGAGGTTGCCAGTTCCCACGTGCCTTCTCTATC[C/T]TGTGGCCATCACGACGGCCAGCGACACAACGACACCACATGGCTTGCTAAT |
| 7 | 7761075 | G | | 24.64 | GAGATGAGGTTGCCAGTTCCCACGTGCCTTCTCTATCTTGTGGCCATCA[G/C]GACGGCCAGCGACACAACGACACCACATGGCTTGCTAATACTCTCTTCCCG |
| 7 | 7764037 | A | | 24.59 | TGTTATAAGGAGTTGGTCATCGTGGGAAATGATGATCTTGTCAATGTAC[A/C]ACATGAATCCTCAATCTGAGGGAAGAAAATTATGTCCACAAATAATATGTA |
| 7 | 7773624 | T | | 27.54 | AACCTGCTGTTGCAATACCCGTTGATTGGTGAACAACTTACAATTTCCT[T/C]ATCAACTTTCAAAAGAAATCATGGTTTGATCAAGTTCACGTCGGGAAAAAC |
| 7 | 7773756 | A | | 27.94 | CGGCTGCCGTCCCCGACGGAAACGGGGCCACCTCCCCGTCCTTCATGCT[A/G]TACACGCCGGAGTGGAGCTCCTCGCTGGAGTCGTTGTTCGACTCGTCACGC |
| 7 | 7774537 | C | | 27.27 | AAGCACATCAAGCTACACCATCTTCACAAGCTGGAGCAGATATGTGAGG[C/T]CAAATTGACTGCACCTGCGTTGCAGTCAGTCGGCATCAGGGACTGTTGGGG |
| 7 | 7774540 | G | | 25.84 | CACATCAAGCTACACCATCTTCACAAGCTGGAGCAGATATGTGAGGTCA[G/A]ATTGACTGCACCTGCGTTGCAGTCAGTCGGCATCAGGGACTGTTGGGGTCT |
| 7 | 7779595 | T | | 34.78 | CACAGCACCAGAGCGGAAAGCGGTGATGAACGCCTTCCCGACACCCTTC[T/C]TTGCCTCGAGAGTGGTCCTGGCATTCGCGTAGGTCGCGATCTTCATTCCGA |
| 7 | 7784309 | G | | 25.26 | TGGCCCATTGCTGTGTTGTTTAGCAGAAGGTTGATTTTAGATATTTGGC[G/A]GAAGGTTGCATTTTTTTTTAAGTAACAGAAGCGGACGTTTTGGGCATTGCG |
| 7 | 7784482 | C | | 25.00 | CCGTATTGGAGATGCCCTAAAAGAGCAGTGAAAACAGATATATTGCAGC[C/T]TGCAAATTCCAAGATAAAAAAAGCCTAACCTTCTCCAGTCGTAGAGCAGTT |
| 7 | 7785831 | G | | 25.00 | CTAGTTTGTACACTGCTTCAGAATCGCAAAACGGGCACCGGAGAAGCTC[G/A]CCTTCATCATCTACAGTTGATGACACCGTTGTAATTTTGCCGACCAGGTGG |
| 7 | 7786178 | T | | 33.33 | GTTAGCGAGATATTCGCACGACTCGGATGTCGTCCATCTCAAAATTGTG[T/C]GTTCGCGCGCATCCGAGGCGGTAGACTTCGCGATTTGCGGATCTGCTGTTT |
| 7 | 7804104 | C | | 26.79 | ATCAAGTATGTCGAAGAAGGAAAAGAAATCACACAATTACTGCACGGAA[C/T]ATTTTAGAGTTGGCTATGCGAGGTTTCGGGGCCAAAGGATTTGACAAGATA |
| 7 | 7804162 | A | | 32.39 | GTTGGCTATGCGAGGTTTCGGGGCCAAAGGATTTGACAAGATAAGGTTG[A/G]ATTACGCCAGATTCACATAATGAAACACTGAAGTGATGTGCTGGTGCGGGC |
| 7 | 7804226 | T | | 41.89 | ACATAATGAAACACTGAAGTGATGTGCTGGTGCGGGCTAGTCACCAGAG[T/C]TGGAAAGGGTGTCCGCTAACTGAGGTTAAACTGACAAACAACTTACATTAT |
| 7 | 7804251 | A | | 42.67 | GCTGGTGCGGGCTAGTCACCAGAGCTGGAAAGGGTGTCCGCTAACTGAG[A/G]TTAAACTGACAAACAACTTACATTATCTCGCAACCGACGTTTGCTAAAGTG |
| 7 | 7808033 | C | | 24.14 | CTCCAATCGTTTCAAGTTCTCTTCGATCATGGCCACCTACCCTTGCATC[C/T]CCAAGTGCATAATACATGAACATTTCGAGGAACACTTCATCATCCAGGTCA |
| 7 | 7810701 | A | | 42.86 | TTATTTTTGACATCACAGTGGACCCTGGTAAGTAAAGTCGGACAAAGGG[A/C]AAAACACTAGTGCAGCATGTAGTTGGGCTCGGTTGTTTGTTATTGCTCTTC |
| 7 | 7810805 | G | | 42.03 | ACTGGGATGAGGGCAGCGCTAAGCTGAGAGGTCCATCCATCCATCAACA[G/A]GGGGATCAACCTGCAGATCCTCTGATTGAGGCAACTTCATCAGATGGATGC |
| 7 | 7813459 | T | | 33.33 | CAGCTCTCCCTTTTCCATTGAAGTACAAGTTCTGTGAGTTTTTCCTTGG[T/C]TGCAAGATTGACTTGAAGAGCCTCCTCCTTGCTCTCAACATTCTCAAGACC |
| 7 | 7814284 | C | | 33.33 | GGTTCTGGAATGCGAAAGTGATCATGATCCCACACCAGTACTAGTTCTG[C/T]GAGTCTTTCCTTGCCAGCAAGATTGATTTCAACAGCCTCTTCCTTAGTCTT |
| 7 | 7814309 | A | | 34.15 | GATCCCACACCAGTACTAGTTCTGTGAGTCTTTCCTTGCCAGCAAGATT[A/G]ATTTCAACAGCCTCTTCCTTAGTCTTAACTGCTCAATACCATGGATCTGCA |
| 7 | 7814346 | G | | 30.49 | GCCAGCAAGATTGATTTCAACAGCCTCTTCCTTAGTCTTAACTGCTCAA[G/T]ACCATGGATCTGCAGCTTGCCTTGAAGTTTGTTTAGGTGTTTCAACTGATG |
| 7 | 7815762 | T | | 23.88 | GCATCCCAGCTGCATCTGCTGAACTTAAGCTCTTGCAGGTTCTTTGGGC[T/A]GACTTTATGCTTACCCATCAGCCAACTCGGTATCTTCCTACCTTGGTAATT |
| 7 | 7815764 | C | | 23.88 | ATCCCAGCTGCATCTGCTGAACTTAAGCTCTTGCAGGTTCTTTGGGCAG[C/A]CTTTATGCTTACCCATCAGCCAACTCGGTATCTTCCTACCTTGGTAATTCC |
| 7 | 7821760 | T | | 27.40 | ATAGCGACTCAAGATGAATGAAAGCCCCAAGGTCAGGAGCAGGTCCCAG[T/C]TGGGTCCATCTTCTGAATGTAAGTTCTTGCAAGTTCTTTGGCCTGCAGGAT |
| 7 | 7821764 | A | | 27.54 | CGACTCAAGATGAATGAAAGCCCCAAGGTCAGGAGCAGGTCCCAGCTGG[A/G]TCCATCTTCTGAATGTAAGTTCTTGCAAGTTCTTTGGCCTGCAGGATAAAT |
| 7 | 7824892 | A | | 30.43 | AACGCACCTGATGTTCTGGTCTTTGCATCTTTGGTGCGGGATTTATGAA[A/T]CCAAATCTTGGTTCATGGACTTACTAGTTCTTTATTTTCTTAGAACTAGCT |
| 7 | 7835645 | C | | 25.49 | GAAGAAGGTTGCCTGGAGTGTGTCTGCCATTGGCCGCTGGGTGGATGGA[C/G]GAGCTGGATCTGCGCGGCAGGCACGCGCGTCACGTGCCGCGTCGCACGGAT |
| 7 | 7837901 | G | | 35.11 | AAACCAAAACCTTAGTTTCAATGCCGGCTTCGATTCTCCCGTGCTTGAC[G/A]TACAAGTTTGTGCAAATGGCCAGTTCAGTGTTAGCGCACTTTGTGCTGAAG |
| 7 | 7839006 | T | | 28.05 | AACCCACAACCAACCCTTCCTCCTTCCTCAAGCGCGTCAGCTCCTCCGC[T/C]CCGGCGACCCGAGCGAGATGGTTTCCGCCGACGCAGCCCGCAACGTCGTCG |
| 7 | 7839011 | T | | 25.00 | ACAACCAACCCTTCCTCCTTCCTCAAGCGCGTCAGCTCCTCCGCCCCGG[T/C]GACCCGAGCGAGATGGTTTCCGCCGACGCAGCCCGCAACGTCGTCGGTATT |
| 7 | 7845702 | A | | 23.88 | TAAAAAACTAATAGAATTAGGATGGGGACAATGTTTTGTGGAAAAGGTC[A/G]TTAACAGGACAGTATAACCAACATTAACTTGAATGTCGATGTAATACGGAA |
| 7 | 7849150 | G | | 30.53 | TTGCACCATAATATGCATCATAGGATTGTGACGGGTCATGAGGGAGGGG[G/A]TTTTTCCATTTGGGACTACCAGAAGCAGGTATCATTACTGCTACTTTATAG |
| 7 | 7852561 | T | | 36.73 | ATATGTGCACTGGAAAAGGAGAACTACCTGCCTGTCATGTCTTTCCCTC[T/G]GCTGATGCGTTTCTCAGTGGAGTATCTGTTGCTTGGTTTTCGTTATGTGCT |
| 7 | 7854086 | A | | 30.77 | TGTAGTAGGCATTCATGCTAACCACATAATTATTATAGTGTGAACTCTC[A/T]TGACCAATGCAGGGCAGAGTGATTCATAGCTCCTCATGTGAATGGCAACAA |
| 7 | 7862823 | T | | 31.18 | ATAGGAGAATCAAGTGTTAGCTCTAATCTTGATTTGTATCTGCATACTT[T/A]GTTGTTTTTGTCATGCATGTGACTTAGATGGCCTAGGGAGTGATAGGCAGG |
| 7 | 7872926 | C | | 32.17 | GGTTTGCACTCTATTAGTTCAATAAATGTTGTAGATCAACATGATAATC[C/A]AATGGTGGATATTCTGTGGCAACCAACTGAACCTGCTATCCCAGCTGTAGA |
| 7 | 7887829 | T | | 31.40 | CTGATCTTGTGTGTCAGCGCCCCCGACAGTGGGGATCCACAAGGCCACG[T/C]TGTCCCCACTCATCAGGCTGCACCCAGGCCCACTCGACCCCATCAGCCCAA |
| 7 | 7888452 | T | | 40.78 | TTCCCTCGCATCCATGGCACGAGCAAGTAAGCCTGAATAAACAGCCTTG[T/G]CACGATCAGGCAACTCTGAAAAATCACGACGATCTCGAGCACTTTGGACGA |
| 7 | 7888482 | T | | 38.33 | GCCTGAATAAACAGCCTTGGCACGATCAGGCAACTCTGAAAAATCACGA[T/C]GATCTCGAGCACTTTGGACGAAGTTGAGGTAATCCTGCGTGTCATAACAAA |
| 7 | 7888523 | A | | 31.08 | AATCACGACGATCTCGAGCACTTTGGACGAAGTTGAGGTAATCCTGCGT[A/G]TCATAACAAAGCTCCCGAACTATCTTCATCCACCACCTGGCCATGAAGCTA |
| 7 | 7888746 | C | | 28.81 | GAGGGCCGAGGGACCCAGCATAACAGTAACAGGAGCTTGCATCGCTGCT[C/T]CAAGATCAGGGTCCGCCGCTATCCCCGCCCTTAGGAGCGCGGCTTCTACAT |
| 7 | 7889372 | A | | 31.15 | CCTCTCTCGTCCATTTCAAGATCTTCTTTCAGGTGTTGGAAAGACAACA[A/G]CTGCCAGAACCTTGTATCACAAGCATGGAGGGAAATTTCAGTGCCGGGCTT |
| 7 | 7889373 | A | | 30.65 | CTCTCTCGTCCATTTCAAGATCTTCTTTCAGGTGTTGGAAAGACAACAG[A/C]TGCCAGAACCTTGTATCACAAGCATGGAGGGAAATTTCAGTGCCGGGCTTT |
| 7 | 7889392 | T | | 34.25 | ATCTTCTTTCAGGTGTTGGAAAGACAACAGCTGCCAGAACCTTGTATCA[T/C]AAGCATGGAGGGAAATTTCAGTGCCGGGCTTTTGTAAGTGTGTCTCAGAAT |
| 7 | 7890168 | A | | 37.65 | GTGAAGGTTTGCCATTAGCAATTGTAAGTATAGCGGTCTGTTAGCAAGC[A/G]AATTAAGCATCGTCATGGAAGATTGGAGGCACATACAGAATTCTTTTTCCT |
| 7 | 7890180 | A | | 34.21 | CATTAGCAATTGTAAGTATAGCGGTCTGTTAGCAAGCGAATTAAGCATC[A/G]TCATGGAAGATTGGAGGCACATACAGAATTCTTTTTCCTCTACTTCCGAAG |
| 7 | 7890184 | A | | 32.00 | AGCAATTGTAAGTATAGCGGTCTGTTAGCAAGCGAATTAAGCATCGTCA[A/T]GGAAGATTGGAGGCACATACAGAATTCTTTTTCCTCTACTTCCGAAGGGAT |
| 7 | 7890186 | C | | 31.17 | CAATTGTAAGTATAGCGGTCTGTTAGCAAGCGAATTAAGCATCGTCATG[C/G]AAGATTGGAGGCACATACAGAATTCTTTTTCCTCTACTTCCGAAGGGATGA |
| 7 | 7890212 | G | | 28.05 | CAAGCGAATTAAGCATCGTCATGGAAGATTGGAGGCACATACAGAATTC[G/T]TTTTCCTCTACTTCCGAAGGGATGAACGATGTTTTAAACCTTATCTACAAT |
| 7 | 7890316 | T | | 24.84 | CTTCCACCTGGTTTGAGGACATGCTTGCTATATCTGAGTATGTATCCAC[T/A]GGGCTACGTGATGAAGAAGGCTGAGTTGGTGAGGCACTGGGTAGCCGAAGG |
| 7 | 7890496 | C | | 32.79 | GTCCAAGCAGTGGACACCAATCATATTGGTGAGGTGTTGTCATGTACGG[C/T]TCACCATTTGGTACTGGATTTTATTAGGTCCAAATCCTTGGATGAGAATTT |
| 7 | 7899098 | A | | 33.33 | TGCTCGATTGGTTTGCTTGGGGTGGCAGTGACGGGGTGCCGGAAGCTGC[A/G]GGACACGGAGTTCTTCTCGCGCCAGGACCCCTACGTCATCGTCGAGTACGC |
| 7 | 7903425 | G | | 35.19 | AACAAACTTTAGCATTTATCTTCAATAACACATTAATCAGGTACTGATC[G/A]TTGACTTTTCTTGGAGGAGCTAGACATTGTGTGAAGATGCCGCATTCAGCA |
| 7 | 7903603 | C | | 36.54 | TAACGACAACCGAAAACATGAACCAACCATAAATTTCACAGTTTTTTCT[C/G]TCAGGAAGAAGGCACAGGAGGAACTTGGGGTCTCCAGGTGGATTGGAATTT |
| 7 | 7903615 | A | | 39.29 | AAAACATGAACCAACCATAAATTTCACAGTTTTTTCTGTCAGGAAGAAG[A/G]CACAGGAGGAACTTGGGGTCTCCAGGTGGATTGGAATTTATCTTAGCAAAC |
| 7 | 7921956 | G | | 23.36 | GCAATTAGAACATCACTAGTAGGTGATTGGTAGCAATTAGAACAACACT[G/A]GTAGGTGATAAGTACCAATTAGAACATCCATACAAGAAGATAAGTAGTAAT |
| 7 | 7921957 | A | | 23.36 | CAATTAGAACATCACTAGTAGGTGATTGGTAGCAATTAGAACAACACTA[A/G]TAGGTGATAAGTACCAATTAGAACATCCATACAAGAAGATAAGTAGTAATT |
| 7 | 7922740 | A | | 29.46 | TATCTATATCTCTCTCTTTCTCTTGATCTTCTTCAGCACGTATCTTCAA[A/G]GTTTGAATAGTAAACCAAGTAAATTATATGACATGCAAGGACAATACAAAC |
| 7 | 7923905 | C | | 37.04 | TCAACAAGTCTTTTTTTTGACAGAAAATAGTGAACTGGAATAGTTCATA[C/G]AAATTGAAACCTGTCCAACCTGAAATTCATAGCGAAGTTGCCCTTTACAAT |
| 7 | 7927906 | T | | 23.66 | TCTCTAGATCAAGACTGCTACTAGGCGCTACTACTTGGATGCCAAGGAA[T/C]AGAAGGACGGTGCGACCAACGTGCTCCTCGGTAACATCGTGAGTGCTCTCC |
| 7 | 7933799 | T | | 30.00 | ACGCTCTGAAGATGACCTGGAAAAAGTTTGTGATGGTTTGTCTGTTAAA[T/G]GCGACGTCATTTCGACAATTCCATAAAGCTCAAAGTAATGCACATGCTCCC |
| 7 | 7933837 | G | | 30.08 | TGTCTGTTAAAGGCGACGTCATTTCGACAATTCCATAAAGCTCAAAGTA[G/A]TGCACATGCTCCCACTCGAATATGTGCCGCGGTCCGAGGCGCCACCCCAGC |
| 7 | 7933855 | A | | 24.46 | TCATTTCGACAATTCCATAAAGCTCAAAGTAATGCACATGCTCCCACTC[A/G]AATATGTGCCGCGGTCCGAGGCGCCACCCCAGCCAACCAGTTATCAAAAAT |
| 7 | 7935161 | C | | 29.09 | GACCCAGTAGTCCACATTATAAGTCCGTTTGTTTACTGTCAGTGAAGAT[C/G]ATCAATGAAACCGAGCTGCTGTTTATATTCCCAAATCAGAGCCGAGAATTT |
| 7 | 7935214 | G | | 28.33 | AATGAAACCGAGCTGCTGTTTATATTCCCAAATCAGAGCCGAGAATTTT[G/T]GTTGCTCAAATTGCCTGAATAAATGGTAGTAATCTCACCACCTCAAGAAAA |
| 7 | 7935237 | T | | 25.00 | ATTCCCAAATCAGAGCCGAGAATTTTTGTTGCTCAAATTGCCTGAATAA[T/A]TGGTAGTAATCTCACCACCTCAAGAAAAGTGGTTATCACAACAACAACATG |
| 7 | 7936900 | G | | 32.00 | TGGTGTTCCAATGGCAGCGGTGGTGGCCACCATGGTGGTCGGACCACTG[G/C]TCAAGATCGTGATGGGGAAGGCGTCCAGCTACCTCCTTGACAAGTACAAGG |
| 7 | 7937040 | T | | 28.91 | GCAAATTGCCTGCCATCCTGGACATCATCACCGATGCCGAGCAGGCGGC[T/A]ACCCACAGAGAAGGGGCAGCGGCCTGGCTCCAGGCCATCAAGAAGGTGGCT |
| 7 | 7937151 | G | | 26.09 | ATGAAGTCTTCGATGAGTTCAAGTACGAGGCACTTCGTCGTGAAGCAAA[G/A]AAGAAGGGGCACTACAAGAAGCTTGGCTTCCATGTGGTAAAACTCTTTCCA |
| 7 | 7937157 | A | | 23.00 | TCTTCGATGAGTTCAAGTACGAGGCACTTCGTCGTGAAGCAAAAAAGAA[A/G]GGGCACTACAAGAAGCTTGGCTTCCATGTGGTAAAACTCTTTCCACTCACA |
| 7 | 7938413 | A | | 29.49 | CTATTCCAGAACTACATGTAAAATCCATGACCTTATGCATGATGTTGCA[A/C]TTTCAGTAATGGAAAAGGAATGTGCCTTGGGAATTGAGGAACCATACAAAA |
| 7 | 7946721 | T | | 25.00 | CAGGAGCAAATCAGCAGAAGAACATGGAGCTCCTCACATTCTTGTGCAG[T/C]CGAGGCAGCTCCGGCACCGGAATGCTGCCCGCCGATGTGTGCCCCCCTCCC |
| 7 | 7950866 | T | | 41.94 | GGAAACCTAGTTAAGCATGCTTAACTAGGTTTCCATTGAGGCGGAGGAA[T/C]GGTGACAACGGAGACACTGATACTGTTGAAATAACAGTCTATGATTACTTC |
| 7 | 7954947 | G | | 34.38 | TGCCATCATCAACTGAAGCTACAGAACCTTTTGCATGTAATACAATGAA[G/C]TACCAGGTGACGTCTAGAATATCACTTAGCGGTGTAGGGATTTATGTCACA |
| 7 | 7958838 | A | | 24.19 | GACAAGTTTGCCATCTGACTTGGAACTGAACAGACAAAAGATCAATTCT[A/G]TGCCCCAGTGTATAGAACTACAGCATGAATAATACTGGAGTACAGAATGCA |
| 7 | 7964544 | T | | 33.33 | GAGCTCCGGAGGACAAGAGAACGAAACTTCACGGCGGAGAGGAAGGAGG[T/C]GGTGGGTCTCGTGGCTGACGAGGGAGGGAGGGAGAGAGGATGGAGGAGGAC |
| 7 | 7970083 | A | | 38.18 | AGATTCCTCTTTCCACAACGAGTACCCTTAGATTACACCTTGCCTTGCT[A/G]AGATTTAGGTGATGGTTGCTAGGCCGCGTACAGGTTCAGATTCAGAAAGTC |
| 7 | 7970771 | C | | 23.21 | GCGCGTCCGTCCTCGATGCGTATCCGCGTATGAAGGCCTGGGTTGATGG[C/G]ATGATGGATCGGCCGAGTGTGGAGAAGGTCATGGAGCTTATGGATGCGTCG |
| 7 | 7972515 | T | | 39.78 | ATCTTTCTCCTTCACTGAAGCTGCTCCCGAACGCCACTCGGGATATCAC[T/G]TCGCCCGTGAGGTTCTGAAACTCTGGCCACACATCTATCTCATGCACATCA |
| 7 | 7972988 | A | | 23.31 | GAGTTCAATTAGCATTAGTATAAGCATGATCAAGGTTGCCGGATGATTA[A/G]TACGTCCAGGTTCTAGGCAGCGTCCGAGTCCTAGTAAGCTTACTAGCTGTG |
| 7 | 7973064 | G | | 34.07 | AGTCCTAGTAAGCTTACTAGCTGTGTCTTAGTTGGTTTACTTTCCAAGT[G/A]GTGGTACGTGTCCGAGTGTGTAACGTGGTGTGCAACCTATTACGAATAGGT |
| 7 | 7973128 | T | | 33.96 | AGTGTGTAACGTGGTGTGCAACCTATTACGAATAGGTTTGGGTTCCCTT[T/A]GGTCGGCTGTTGATGCATATATATATGCACAGCCATGGCGTGAGTTTGTAA |
| 7 | 7973151 | G | | 42.17 | TATTACGAATAGGTTTGGGTTCCCTTAGGTCGGCTGTTGATGCATATAT[G/A]TATGCACAGCCATGGCGTGAGTTTGTAACACCGTGAGTTGAGAAACAAGAA |
| 7 | 7987827 | T | | 28.57 | CAAAATGCCTATCACATTCTATGACATTCAAAATGCCTGCTAAGTATCC[T/C]GTGTATTTTGCTCCTTTGGATATGTTGCAGTTACGCTGCCCATGCAGATAA |
| 7 | 7987844 | C | | 30.36 | TCTATGACATTCAAAATGCCTGCTAAGTATCCCGTGTATTTTGCTCCTT[C/T]GGATATGTTGCAGTTACGCTGCCCATGCAGATAACTGAACTTTTGGTGCTT |
| 7 | 7987845 | A | | 29.82 | CTATGACATTCAAAATGCCTGCTAAGTATCCCGTGTATTTTGCTCCTTT[A/G]GATATGTTGCAGTTACGCTGCCCATGCAGATAACTGAACTTTTGGTGCTTA |
| 7 | 7988109 | A | | 23.33 | TAAGCTATCCAGTTCTTTTCACTTCCTTTCATTTAGCTTATTTCCTCAT[A/C]TGTCAGGTGAATAGTCTGTAATACCCTTGAATAGTATGGAAGGGGAACAGC |
| 7 | 7997331 | C | | 32.98 | ACATCGGATTGGTCTCAGAAGAGGCCGAAGCCCTGCCGGCCATGGTGGC[C/T]AGAGCAAAGGAGCTGATTGGCGCCGATACACCATGAGTTTGACATGGCCAC |
| 7 | 7997352 | T | | 30.28 | AGGCCGAAGCCCTGCCGGCCATGGTGGCTAGAGCAAAGGAGCTGATTGG[T/C]GCCGATACACCATGAGTTTGACATGGCCACCGTGAATAAGTGCCATCTTGG |
| 7 | 7997361 | G | | 25.23 | CCCTGCCGGCCATGGTGGCTAGAGCAAAGGAGCTGATTGGCGCCGATAC[G/A]CCATGAGTTTGACATGGCCACCGTGAATAAGTGCCATCTTGGTGGCGTGGT |
| 7 | 7997408 | C | | 24.30 | ACACCATGAGTTTGACATGGCCACCGTGAATAAGTGCCATCTTGGTGGC[C/G]TGGTGGTTGGGTAGTATCCATCCACTGTTGCATGCAGGCACAGACCAGATA |
| 7 | 7997515 | T | | 24.10 | TGTGATCATCAAAATGTACTTGGCGCCCATATGTGGCATGGACCAACTT[T/C]CTTTGGTGTTTCCAATATTTTCTTGTTGCTCCTCGCTCTTCAATGACGTCT |
| 7 | 8006755 | C | | 30.88 | GGAGGAGGTGAAGCGCTTCGTGTACGCCCACTACGACGAGCATCTCAAG[C/G]ATAGATTCTACGCTTCCGACCTCGCCAAGGACCTGCAGCTGCCGGCGGAGG |
| 7 | 8029534 | T | | 40.35 | CACACTCACAGCAGAGCTCATCAGTTTGGTGGCGTCCTCTAGCCATGTA[T/C]TCGAGAAGCTGCTGCAGGCGCTGGATTCCACAAGCCCATACGGCGAGACGA |
| 7 | 8029874 | A | | 35.38 | GTCTTGGCGCCGGTAAGCTCCGACCTGCTCCACCTCATCGACCACGAGG[A/C]ATGGTCGACCACCATCGCAGCCGCGTCGCTGCAAGTCATGTGCCGCCTCGT |
| 7 | 8035489 | A | | 33.33 | GGATTCTAACACCGATGACAATTCCGGATTTCTTCAAGCGTGATGGTCA[A/G]CAGTATTTGATTAGTGGCTCTCGTGACAAGACTGCTAAGGTCGGCTATTAT |
| 7 | 8037698 | G | | 33.33 | CTAATTTTCCAGCTTATTGTGCTCTGGCTTTCCAGCTTTCTCTTCTGAC[G/A]CATTCCATTGAATTATGTATCCTCAGATACTCGTGGCTGTTGTTAGCATCG |
| 7 | 8040766 | T | | 36.36 | GTGTTTTCCTTAGTGTTCCCATGGCGGAGCTGGTGGCCACCATGGTGGT[T/C]GGACCACTGCTCTCCATTCTCAACAATAAGGTATCCAGCAGCCTGCTCGAC |
| 7 | 8040868 | T | | 33.33 | AGTACAAGGTGATGAAAGGCATGGAGGAGCAACATGAGATCCTAATGCG[T/C]AAGCTTCCTGCCATTCTGGACATCATCGCCGACGCCGAGAAGGCGGCATCC |
| 7 | 8040954 | C | | 34.43 | GAGAAGGCGGCATCCCTGAGAAGAGGTGCAGCGGCATGGCTCGAGGCCA[C/T]CAAGAAAGTGGCTTACCAGGCCAATGAAGTCTTTGATGAGTTCAAGTATGA |
| 7 | 8040970 | T | | 32.53 | TGAGAAGAGGTGCAGCGGCATGGCTCGAGGCCATCAAGAAAGTGGCTTA[T/C]CAGGCCAATGAAGTCTTTGATGAGTTCAAGTATGAGGCGCTTCGCCGCAAG |
| 7 | 8041152 | C | | 30.33 | ACAGGATGGGAAGGAAGCTCCGCAAGATTGTGCAGGCCATCGAGGTCCT[C/T]GTGACCGAAATGAACGCCTTTGGCTTTAAGTATGAGGAACAGCCGCTGGTC |
| 7 | 8041610 | G | | 30.53 | ATTCTTCAGAATGTAGTAAGCGGGCAAAGGTACCTCCTTGTATTGGATG[G/A]TTTCTGGGAACGACAGGTTGAGATTTGGAGACAGCTCAAGGCCGCCTTGAA |
| 7 | 8041655 | T | | 31.25 | GATGATTTCTGGGAACGACAGGTTGAGATTTGGAGACAGCTCAAGGCCG[T/C]CTTGAATATGGTGGCATGGGTAGTGTGGTCTTGACAACTACTCGTGACGAA |
| 7 | 8041794 | A | | 35.16 | TCTCAAAGCTTTGGAAGATAAATTCATAAAGAAAATCATCGAGACAACA[A/G]CATTCAGCCGTTTCAAGAAGGCTGAGGAAAGGCCTAGCGAGTTGGTGGAAA |
| 7 | 8042015 | C | | 28.00 | TTTGCACCGAGGAGACATGAATCTTACCAATACTCAAGCTCAGTTACAA[C/T]GACTTGCCGTCGCATATGAAGCAATGCTTTGCTTTTTGTGCTGTGTTTCCC |
| 7 | 8042027 | A | | 33.33 | AGACATGAATCTTACCAATACTCAAGCTCAGTTACAATGACTTGCCGTC[A/G]CATATGAAGCAATGCTTTGCTTTTTGTGCTGTGTTTCCCAAGGATTATGAG |
| 7 | 8042311 | T | | 23.08 | ATGGTTGATAAGATTGTGGAGAGATGTGCTGGCTCTCCTTTAGCTGCAA[T/C]AGCACTAGGCTCTCTACTGTGTACCAAGACCAGTAAGGAAGAATGGGAGGC |
| 7 | 8042917 | A | | 29.31 | AATTGAATAGTTCTCTGGAGAACAGCTCTCCAGCCATCCAAACACTTTT[A/G]TGTGATAGCTATATGAGTAGTTCATTGCAACATCTATCAAAATACACCTCT |
| 7 | 8042925 | C | | 24.53 | AGTTCTCTGGAGAACAGCTCTCCAGCCATCCAAACACTTTTGTGTGATA[C/G]CTATATGAGTAGTTCATTGCAACATCTATCAAAATACACCTCTTTGCAAGC |
| 7 | 8043524 | C | | 26.87 | ATAATGATGGGATGCAAGGTGAGGTGATGATGTTTCCTCTACTTGAGAA[C/G]CTGCATATTAGCGACAGTGTAAAGTTGAAAGCATTGCCAGGAAAACCGACC |
| 7 | 8043605 | T | | 36.00 | CATTGCCAGGAAAACCGACCTTCCCTAAGCTTCAGAATGTTCGTGTTGA[T/G]AAATGTCCAGAGTTGACAACTGCAGCTAAATCACCAAAGCTCGGTGTATTG |
| 7 | 8043737 | T | | 29.29 | TGTGGGTAGCGAGACATCTAACTTCATTGACCAATCTGGAACTGACTAG[T/C]ACTGAACATAGTACAGATACAACCTCGATGGGGGCTGAGAATAGTTTGAGG |
| 7 | 8043770 | A | | 28.92 | ATCTGGAACTGACTAGCACTGAACATAGTACAGATACAACCTCGATGGG[A/G]GCTGAGAATAGTTTGAGGGAAGTGGTGAACATCAAGGAGAATGGGAATGCT |
| 7 | 8043787 | A | | 37.50 | ACTGAACATAGTACAGATACAACCTCGATGGGGGCTGAGAATAGTTTGA[A/G]GGAAGTGGTGAACATCAAGGAGAATGGGAATGCTCAAAACTTTCCTTTAGA |
| 7 | 8043869 | C | | 41.67 | CTCAAAACTTTCCTTTAGAAGTCTTGGTGTTAAGAGACTTTAAGTCAGG[C/T]GTACGTGTAACAGAGCTATGCGCATGCTTTGTACACCTTCAAGATTTGTCA |
| 7 | 8043870 | A | | 33.87 | TCAAAACTTTCCTTTAGAAGTCTTGGTGTTAAGAGACTTTAAGTCAGGT[A/G]TACGTGTAACAGAGCTATGCGCATGCTTTGTACACCTTCAAGATTTGTCAA |
| 7 | 8043914 | A | | 30.16 | CAGGTGTACGTGTAACAGAGCTATGCGCATGCTTTGTACACCTTCAAGA[A/T]TTGTCAATTATGAGGTGCCATGAGCTCGTCCACTGGCCAGAAACATTGTTC |
| 7 | 8044204 | T | | 37.70 | ATGTACATTGATAAATGCAGTAAGCTTGAGTCCACATCCGGCAGGAAGC[T/A]GAAGCAGGGACAGTCAGTATCATCGACTCATCAAGGGTCATCCAGTATAGA |
| 7 | 8045073 | T | | 40.00 | GCAAGACCCTTCACGATCAGTAATTGTTGTAGCTGGGATCAGGTGCGCA[T/C]AGGAAGTTGTTGCCATGCCGAATCGGGAGATCAGTAATCAGAAAGCACTCC |
| 7 | 8045490 | G | | 31.00 | TATCTGTAGCAGGTAGTGAGCCTAATTGTAGTAATGTTGGAGAGCTTGG[G/A]AATTTAAACCTTGGTGGTCAACTAAAGCTACGTAATCTGGCAAACGTGACA |
| 7 | 8045590 | C | | 36.71 | AGAAGTGGATGCAAAAGCAGCAAACCTTGTGAACAAGGAGCTAAGAGAA[C/T]TGAGATTAACATGGACCTTCAGATGGAATTATTTTCAAGGTAATACTAGCT |
| 7 | 8055746 | A | | 39.62 | CGAAAACATGGCGAAAGAAATCATGTCTTCCTTTCCTCCTTAAAATTCT[A/G]CGAGCTGCAACCTACGCCTTGGCTGTCACAGGAGTAGCAGCAGTAGAACCG |
| 7 | 8055749 | G | | 38.89 | AAACATGGCGAAAGAAATCATGTCTTCCTTTCCTCCTTAAAATTCTGCG[G/A]GCTGCAACCTACGCCTTGGCTGTCACAGGAGTAGCAGCAGTAGAACCGCCA |
| 7 | 8056144 | G | | 40.38 | GGTTCCTGCATGAAGGGATTCCAATTAATTATGATATATATGGTGCCAA[G/A]TTTGAAAACACTGATAACAATTGAACAAGCAAAGTCAAATACAAAATTAAC |
| 7 | 8062176 | A | | 27.69 | CTGGAGAAGGAAAAAGGCCATGTGAAGCCCACTAGCGGCCAGGTAGGGC[A/G]TTGCGTTCTCCTGTACGTGGATTTCACTCAAAAATAAAAAAGCTTGTACGT |
| 7 | 8062237 | T | | 25.00 | TGTACGTGGATTTCACTCAAAAATAAAAAAGCTTGTACGTGGATTAGTA[T/C]CCTGAAATTAGTCGCCACCGTTCTGAGCCCTTGCCCGCCCCGCCGCCTCTG |
| 7 | 8062254 | G | | 25.45 | CAAAAATAAAAAAGCTTGTACGTGGATTAGTACCCTGAAATTAGTCGCC[G/A]CCGTTCTGAGCCCTTGCCCGCCCCGCCGCCTCTGCTCTCCCTGCCCTACTG |
| 7 | 8062261 | C | | 24.07 | AAAAAAGCTTGTACGTGGATTAGTACCCTGAAATTAGTCGCCACCGTTC[C/T]GAGCCCTTGCCCGCCCCGCCGCCTCTGCTCTCCCTGCCCTACTGCGATCTC |
| 7 | 8062263 | T | | 24.07 | AAAAGCTTGTACGTGGATTAGTACCCTGAAATTAGTCGCCACCGTTCTG[T/A]GCCCTTGCCCGCCCCGCCGCCTCTGCTCTCCCTGCCCTACTGCGATCTCGA |
| 7 | 8062278 | G | | 30.51 | GATTAGTACCCTGAAATTAGTCGCCACCGTTCTGAGCCCTTGCCCGCCC[G/C]GCCGCCTCTGCTCTCCCTGCCCTACTGCGATCTCGATCCTATTCGTACGGC |
| 7 | 8062723 | T | | 38.60 | AGAAAAGAGAGATGAGGTATTCCGATCTCAAGAAGATTGACCGAGGTGT[T/C]CGTCGCCACTTCCATGACGACATAACCGTCATCATACTCTACCTCGACTCG |
| 7 | 8064786 | C | | 25.49 | GAAACATCAAACCATGATGCCATGCACAGTTGTAGTAGAATACGCCAAA[C/T]ATTATGGAAGTATACTTGTCAGGTCAATTTGTTCACATGGGAATGAGAAAC |
| 7 | 8064801 | T | | 32.76 | GATGCCATGCACAGTTGTAGTAGAATACGCCAAATATTATGGAAGTATA[T/C]TTGTCAGGTCAATTTGTTCACATGGGAATGAGAAACTTACACTACGAGGGC |
| 7 | 8067912 | T | | 31.31 | GGCCTGTACTACCACATCCCTAAATCTAATCCCCTCGCCCGTTGGTTTT[T/C]GTCTAGATAGAACAAGATCTGGCCCTAACCCATGCCCGTGCGTGCGTGCCT |
| 7 | 8068065 | A | | 30.33 | TGGTGGCCGTCGTGCTCGGCGTGCTCCTCGGTGCCCACACCCACGAGAA[A/G]CGCTCCATGATCGTCGGCATCCTCGGCGTCATCTTCGGAACCATCATGTAT |
| 7 | 8075336 | G | | 29.82 | TCTACTCCGCCTTCACCGACATCGTGAATCACGCCCCTGGTAAGTGTGG[G/A]TGTTGCAAGTCAAATCTGGTTTCTAATGTGTCAATTCATCTATGTAACTAA |
| 7 | 8075387 | T | | 31.58 | GTTGCAAGTCAAATCTGGTTTCTAATGTGTCAATTCATCTATGTAACTA[T/A]TTTTTTTTCGTGCATGCAGGTGAAGGGTCAGATCGGTCCAAGGACTTCAAG |
| 7 | 8075780 | G | | 42.11 | GCGTATTTAGGGGTGAGCTTCAGAGGTATTATTTTCGTCATGTTTAGCT[G/T]TACCTTTAGTCTAGTAGTAGTCTTTATAGGTGAACTTGTTCCAAATTGCTT |
| 7 | 8075781 | A | | 42.67 | CGTATTTAGGGGTGAGCTTCAGAGGTATTATTTTCGTCATGTTTAGCTT[A/T]ACCTTTAGTCTAGTAGTAGTCTTTATAGGTGAACTTGTTCCAAATTGCTTA |
| 7 | 8077953 | T | | 27.42 | CCACCACCACCACTCACCTTCTGCTGACCGCTTGCCACGGTCTCCTTGT[T/C]GTCCTGAAGCTGCTGCCCCCATTACAAAGAGAATGCGGCCTATCTCAAGAC |
| 7 | 8082727 | T | | 23.00 | TGTCCTGTTGCTAATGGACTCCTCCGGCTCTGGCTGCTGGTCCAGTTGC[T/C]TTCGACAGCTCTCCCGCACCTTGGTTCGCTTTCGCCCGGACAACCGACCCG |
| 7 | 8082732 | G | | 23.96 | TGTTGCTAATGGACTCCTCCGGCTCTGGCTGCTGGTCCAGTTGCCTTCG[G/A]CAGCTCTCCCGCACCTTGGTTCGCTTTCGCCCGGACAACCGACCCGGCTCT |
| 7 | 8082743 | A | | 26.44 | GACTCCTCCGGCTCTGGCTGCTGGTCCAGTTGCCTTCGACAGCTCTCCC[A/G]CACCTTGGTTCGCTTTCGCCCGGACAACCGACCCGGCTCTCCTTTCGGACA |
| 7 | 8082744 | T | | 26.44 | ACTCCTCCGGCTCTGGCTGCTGGTCCAGTTGCCTTCGACAGCTCTCCCG[T/C]ACCTTGGTTCGCTTTCGCCCGGACAACCGACCCGGCTCTCCTTTCGGACAC |
| 7 | 8082760 | G | | 31.94 | CTGCTGGTCCAGTTGCCTTCGACAGCTCTCCCGCACCTTGGTTCGCTTT[G/C]GCCCGGACAACCGACCCGGCTCTCCTTTCGGACACAGAGGAACAACCTGGT |
| 7 | 8082765 | A | | 33.82 | GGTCCAGTTGCCTTCGACAGCTCTCCCGCACCTTGGTTCGCTTTCGCCC[A/G]GACAACCGACCCGGCTCTCCTTTCGGACACAGAGGAACAACCTGGTACTCC |
| 7 | 8083215 | C | | 41.11 | ACCTGAACAGACCAACAATTGCAAGGGCTTCAATCTGATGTGTTGTACC[C/A]CACGGTATTCCTCTACCTGCGTATTCCCACGAATCCTATTTCTTCCATACA |
| 7 | 8083221 | C | | 42.70 | ACAGACCAACAATTGCAAGGGCTTCAATCTGATGTGTTGTACCACACGG[C/T]ATTCCTCTACCTGCGTATTCCCACGAATCCTATTTCTTCCATACATATGTG |
| 7 | 8083245 | T | | 35.96 | CAATCTGATGTGTTGTACCACACGGTATTCCTCTACCTGCGTATTCCCA[T/C]GAATCCTATTTCTTCCATACATATGTGCCTTTCAGGATTCGCTTCTACTAT |
| 7 | 8087030 | A | | 28.79 | CTGCTCGGATCGCCGCCATGCCAGCTTCTGTGCAGATGTTGCGCAAGTC[A/G]GCGGCGTTGAATCCCTGAAAGGCAACGAGGGAGTTCAGAGGAGAAGCTCTG |
| 7 | 8090989 | G | | 30.00 | GGTAGTAGGCTGCGCCATCTCCCCTCACCACCCAGGGATCTACCACGCC[G/A]ACCTTCTCCTCCTTCTCCTTCATGTCTGCCATCCTCTGCAGGTAAGGCTCT |
| 7 | 8094834 | G | | 31.03 | ATCATATTATATATTTGTCGGCTAGCAAAGCATATCGATCTCAAGAGAT[G/A]TCGCGTGATCGCAAACTACTGTGCTATGTGTGTCCGTAGTATGAGTGATTG |
| 7 | 8099795 | A | | 24.68 | CACATCTATAACTCATTCCCTGTGCCTCTTGCGCGGCGGCGGCACAGAT[A/G]TAGGGCAGTGGTGGCGGGTGCATATGTAGGGCAGCGGGAGCGGGTACAACC |
| 7 | 8099799 | A | | 26.99 | TCTATAACTCATTCCCTGTGCCTCTTGCGCGGCGGCGGCACAGATGTAG[A/G]GCAGTGGTGGCGGGTGCATATGTAGGGCAGCGGGAGCGGGTACAACCATCT |
| 7 | 8101078 | T | | 35.00 | GCCCATCAAATTGTAAGTACATAAGCATTCAGTTCTTCTATAATTTGTA[T/C]GACAATACAATTTGGATGTTACATGTAAACAATGTTACCCCTTACAGATAA |
| 7 | 8101293 | G | | 23.08 | ATGAGTACGGAACACAAACCGATGTGCTTGGTCTGTGCTTTGTGTTTCT[G/A]TCACGACCTTTTTTAGAAGGCGCCAAGATGAATGTTAATTCATTAGAGGAG |
| 7 | 8102310 | T | | 32.81 | ACTGAAGGCAGACAGACCTCCAAGCAGCATTCTGTGTCCTCCCAAAGGA[T/C]ATGCAGCAGCTGCTGCTTTCCAATCCCAACGAGCCGTACTTCTTCAAGCGA |
| 7 | 8102321 | C | | 33.87 | ACAGACCTCCAAGCAGCATTCTGTGTCCTCCCAAAGGACATGCAGCAGC[C/T]GCTGCTTTCCAATCCCAACGAGCCGTACTTCTTCAAGCGAGCCAAGAAAAG |
| 7 | 8103385 | A | | 26.04 | TAATATCCTTCGGTGGGTTGTTTGATACCAGTCTACCATTCTCTCTCCT[A/G]TATTTGATAGACGGGAGGGATGCATGCACATGACGGTTGAAGATTTTTCAC |
| 7 | 8104528 | C | | 37.04 | GCTCCATTTTCCCATCAATCCAAATGCTATACCAGGCTTGAGGAAATAG[C/T]CTATGCGTCAATATCTTTGGTACTTTTCCATTCCTTCTAGGTTCATCAAAT |
| 7 | 8107216 | A | | 35.29 | CGGCGCTCGTGTTCCGGTCGAGTTGGTTCCTGTGAGGCCTGAGTGAGTG[A/G]TTTTGGTGTCACCCCTCCTGAGGATGTAGAGAAGCCATGGTAGATCAGAGA |
| 7 | 8107226 | G | | 31.33 | GTTCCGGTCGAGTTGGTTCCTGTGAGGCCTGAGTGAGTGGTTTTGGTGT[G/C]ACCCCTCCTGAGGATGTAGAGAAGCCATGGTAGATCAGAGAAAGTAGAAAC |
| 7 | 8107228 | T | | 31.71 | TCCGGTCGAGTTGGTTCCTGTGAGGCCTGAGTGAGTGGTTTTGGTGTCA[T/C]CCCTCCTGAGGATGTAGAGAAGCCATGGTAGATCAGAGAAAGTAGAAACAA |
| 7 | 8113058 | T | | 32.26 | GCAATTTCTGGAATAGAGCCCTCGAGAGAACTGGACACACGGTGTGAAG[T/C]GGGGGCAGACAAGATACGCGTGGCATGTACCAAGTAAATTGAATAGCCCAG |
| 7 | 8116921 | T | | 28.99 | GAGATGAAACTCACAGAAAAATCTTTCGATGGCGACTGTAATCCGCAAG[T/G]CAAGCGGCCAGGACCATGGGCTGCAGCTAGTCGGCGGCCGTCATCTACACA |
| 7 | 8116945 | T | | 25.64 | TTCGATGGCGACTGTAATCCGCAAGGCAAGCGGCCAGGACCATGGGCTG[T/C]AGCTAGTCGGCGGCCGTCATCTACACACATGTGCACGTCCCAAGCGGCCGT |
| 7 | 8116960 | T | | 26.32 | AATCCGCAAGGCAAGCGGCCAGGACCATGGGCTGCAGCTAGTCGGCGGC[T/C]GTCATCTACACACATGTGCACGTCCCAAGCGGCCGTCTGTATGCAACATCC |
| 7 | 8117194 | G | | 26.00 | CGGCAATCACAAGAGAACTGTTAGATCGGGAGGCCGGCAACGCATAACA[G/T]CCACAACGGCGGCCAGATCAGGATGCGACCACCAGGGAAGACTAAAAAACC |
| 7 | 8117517 | A | | 33.90 | GGTGAGGTTTTGGGGCGGAGGGTGGCGGCGATGAGCGGACGGATGCGGT[A/G]TCGCCGTGCAGTGGCCTGATTTGAAGACGAAGGGGTCGGCGCTTGTGCGGC |
| 7 | 8117793 | T | | 24.07 | GATTTAAGTCGTTTCTCGTTTTTTCGCCATGAGTTCTCCATTCCTTGCT[T/C]AGCTTATGTGGATGTAACTCATGTAAGTCGAGTAGCCATGTTTCGTCACTC |
| 7 | 8124223 | C | | 23.94 | ACAGCCTGTTGAACACTGAACATAAAAATGTCGTACGGTTTCTTGGCTT[C/T]TGTGCTAGCACAGACAAATAGCCATACCAACCGGAGGATCGAAAGAACATA |
| 7 | 8124255 | T | | 40.30 | GTACGGTTTCTTGGCTTTTGTGCTAGCACAGACAAATAGCCATACCAAC[T/C]GGAGGATCGAAAGAACATATTTACGCTGAGGTACGAGAAAGATTACTCTGT |
| 7 | 8124289 | A | | 38.46 | AATAGCCATACCAACCGGAGGATCGAAAGAACATATTTACGCTGAGGTA[A/C]GAGAAAGATTACTCTGTTTTGAGTATATCAGCAACGGAAGCCTGAAAAAAT |
| 7 | 8125000 | C | | 25.00 | TTGTTTGATGTTATAATGCATTGAAATACGACCTTTTATGCAGAGGATA[C/T]TGTGCTCCAGAATATCTACATCAGGGAAAGATGTCATTCAAATCAGACATG |
| 7 | 8139123 | A | | 38.57 | GCCTTGTCATCGTCGGTGCCATTGGTGCTAGCAGCGCTGATGCGCGGCG[A/G]CCGGTGATGCCGGCGATATTCGTGCTGGGGGACTCGACGCTGGATGTGGGC |
| 7 | 8139165 | A | | 30.00 | CGCGGCGGCCGGTGATGCCGGCGATATTCGTGCTGGGGGACTCGACGCT[A/G]GATGTGGGCAACAACAACCACCTGCCCGGAAAGGACGTTCCCAGGGCCAAC |
| 7 | 8145359 | T | | 27.87 | TCAGGATCGTGATGTCTCCTCGTGTTCATTAGACGTTCATCCAACCCAG[T/C]CGTATGTGCTGACAGAATGTGGTACTGAAATAAAGCTTTGGGACTGGGAAC |
| 7 | 8146253 | C | | 32.14 | CAGAAATGACTCTGAATATAAGTTTGGACAGGCAAGCTCGTATGCTCCC[C/T]TAGAAGTACAAGTTATCTATGGACTACACTCCCTAGTTGTCTCCGTCTTAT |
| 7 | 8146263 | T | | 36.36 | TCTGAATATAAGTTTGGACAGGCAAGCTCGTATGCTCCCTTAGAAGTAC[T/A]AGTTATCTATGGACTACACTCCCTAGTTGTCTCCGTCTTATGTTATACACA |
| 7 | 8146269 | G | | 33.33 | TATAAGTTTGGACAGGCAAGCTCGTATGCTCCCTTAGAAGTACAAGTTA[G/T]CTATGGACTACACTCCCTAGTTGTCTCCGTCTTATGTTATACACAGTGGTG |
| 7 | 8146278 | G | | 36.54 | GGACAGGCAAGCTCGTATGCTCCCTTAGAAGTACAAGTTATCTATGGAC[G/T]ACACTCCCTAGTTGTCTCCGTCTTATGTTATACACAGTGGTGGAGCTACAG |
| 7 | 8146683 | A | | 25.71 | ATGCACCATTCAGCTAACCAATGAGACAGACTCTTACATTGCCTTCAAC[A/G]TCGAACATATGAACCCTCTGTCATACTGTGCACAACCGCAGAAAGACATTA |
| 7 | 8147095 | C | | 41.30 | GGCATGTTTAGTTTGCCGAGTTTGAGTGGTATTGTTGCTGATCAGGTTG[C/G]TGTGTATATATGTAGCACTACATCAGTACACAGCAGGGCTTTTTATACCCG |
| 7 | 8149129 | C | | 29.41 | ACATGGGATATGAAGACTGGAAGGGGAAGCGTGGTGCCAACGAGAGGGT[C/T]CCCGGGCTCGTCTCCTACCGCAGATCCGACATCTCCGACATGGAAGACCAA |
| 7 | 8149174 | G | | 35.24 | GGGTTCCCGGGCTCGTCTCCTACCGCAGATCCGACATCTCCGACATGGA[G/A]GACCAAGACGGTTGTGAAAATGTGAGGGATGACGGTGACGATGGTGGTGCC |
| 7 | 8149197 | C | | 40.95 | CGCAGATCCGACATCTCCGACATGGAAGACCAAGACGGTTGTGAAAATG[C/T]GAGGGATGACGGTGACGATGGTGGTGCCCGGTAGCTACAACTGCTTCTTGT |
| 7 | 8149203 | C | | 40.78 | TCCGACATCTCCGACATGGAAGACCAAGACGGTTGTGAAAATGTGAGGG[C/A]TGACGGTGACGATGGTGGTGCCCGGTAGCTACAACTGCTTCTTGTCCCCGT |
| 7 | 8149228 | C | | 33.68 | AAGACGGTTGTGAAAATGTGAGGGATGACGGTGACGATGGTGGTGCCCG[C/G]TAGCTACAACTGCTTCTTGTCCCCGTGGAGTGGCATCCACACAATAATGGT |
| 7 | 8149263 | G | | 35.37 | GATGGTGGTGCCCGGTAGCTACAACTGCTTCTTGTCCCCGTGGAGTGGC[G/A]TCCACACAATAATGGTGTTCCGGTCAGCTACCAGACGAAAAAGGATGGCCA |
| 7 | 8149272 | T | | 28.57 | GCCCGGTAGCTACAACTGCTTCTTGTCCCCGTGGAGTGGCATCCACACA[T/A]TAATGGTGTTCCGGTCAGCTACCAGACGAAAAAGGATGGCCACAGGTGCGG |
| 7 | 8149274 | G | | 29.73 | CCGGTAGCTACAACTGCTTCTTGTCCCCGTGGAGTGGCATCCACACAAT[G/A]ATGGTGTTCCGGTCAGCTACCAGACGAAAAAGGATGGCCACAGGTGCGGCA |
| 7 | 8149300 | A | | 30.43 | CCGTGGAGTGGCATCCACACAATAATGGTGTTCCGGTCAGCTACCAGAC[A/G]AAAAAGGATGGCCACAGGTGCGGCACAGGCTGGGTGGCCAGTGGGCTTTTT |
| 7 | 8149496 | T | | 27.78 | TTTATGCAGATGAATTAAGGGGACTTAAATGGAACACACGTTATGAAAT[T/A]ATTAGAGGCATTTGTGAAGGTTTGTACCATCTGCACCATGAAAGAAAATAT |
| 7 | 8149518 | C | | 30.16 | ACTTAAATGGAACACACGTTATGAAATAATTAGAGGCATTTGTGAAGGT[C/T]TGTACCATCTGCACCATGAAAGAAAATATACCATATGGATATGAAACCTGA |
