# Supplementary material for: Mapping‐by‐sequencing in complex polyploid genomes using genic sequence capture: a case study to map yellow rust resistance in hexaploid wheat
Source: Plant J. 2016 Jul 18;87(4):403–19. doi: 10.1111/tpj.13204 (PMC5026171; doi:10.1111/tpj.13204)
Supplement: Supplementary file 3 [file TPJ-87-403-s003.docx]

**SUPPORTING INFORMATION LEGENDS**

**Figure S1. Seedling screen of 201 progeny lines of a doubled haploid population.** Histogram to show the number of doubled haploid lines with each infection type on a scale of 0-9. Infection tending towards 0 corresponds to a complete absence of sporulation. Highlighted are the respective phenotypes of Avalon and Cadenza parental cultivars.

**Figure S2. Physical positions and genetic marker positions of capture design contigs.** Comparing the physical positions (bp) of 12,900 capture design contigs within the genome B chromosomal pseudomolecules, to their Genome Zipper assigned genetic positions in the A, B and D genomes (cM).

**Figure S3.** **Homozygosity scores calculated for the bulk segregant dataset along each POPSEQ based pseudo-chromosome.** Magenta; Scores plotted for ‘Cadenza specific homoeologous homozygote SNPs’ found in the bulk segregant dataset. Blue; Scores plotted for ‘Avalon specific homoeologous homozygote SNPs’ found in the bulk-segregated dataset. Scores calculated per 500,000bp window along each chromosome at 10,000bp intervals.

**Figure S4.** **Homozygosity scores calculated for the bulk segregant dataset along each POPSEQ chromosomal pseudomolecule.** Magenta; Scores plotted for ‘Cadenza specific homoeologous homozygote SNPs’ found in the bulk segregant dataset. Blue; Scores plotted for ‘Avalon specific homoeologous homozygote SNPs’ found in the bulk-segregated dataset. Scores calculated per 500,000bp window along each chromosome at 10,000bp intervals.

**Figure S5.** **Homozygosity scores calculated and plotted using workflows implemented through iPlant for the bulk segregant dataset along the MIPs based pseudo-chromosomes.** Scores plotted for ‘Cadenza specific homoeologous homozygote SNPs’ found in the bulk segregant dataset. Scores calculated per 500,000bp window along each chromosome at 10,000bp intervals.

**Table S1. Summary of mapping statistics across the pseudo-chromosome reference.** Mapping depth of coverage of the pseudo-chromosome reference and SNP numbers that were identified for the purebred parental lines Avalon and Cadenza plus the bulk segregant dataset. SNPs scored at a minimum quality of 15 and a depth of 30 (for mapping using the POPSEQ chromosomal pseudomolecules directly SNPs were called at a minimum depth of 5). A minimum alternate allele frequency of 10% was used for all SNP calls.

**Table S2. Detailing gene regions within the peak interval 13,650,001-14,150,001bp on the Genome zipper--based pseudo-chromosome 7.** Taken from BLASTN alignments over 1000bp long with an e-value less than 1e-5 and identity greater than 80%.

**Table S3. Detailing homozygous SNP alleles, in 23-43% of reads, in the bulk segregant dataset within the peak interval (13,650,001 and 14,150,001bp).** These SNPs are conserved with unique homozygous SNP alleles, in 23-43% of reads, which were found in the Cadenza parent.

**Table S4. Detailing gene regions within the peak interval 7,650,001-8,150,001bp on the MIPS-based pseudo-chromosome 7.** Taken from BLASTN alignments over 1000bp long with an e-value less than 1e-5 and identity greater than 80%.

**Table S5. Detailing homozygous SNP alleles, in 23-43% of reads, in the bulk segregant dataset within the peak interval (7,650,001 and 8,150,001bp).** These SNPs are conserved with unique homozygous SNP alleles, in 23-43% of reads, which were found in the Cadenza parent.
